# Inhibition of autotaxin alleviates pathological features of hepatic encephalopathy at the level of gut–liver–brain axis: an experimental and bioinformatic study

**DOI:** 10.1038/s41419-023-06022-5

**Published:** 2023-08-01

**Authors:** Ali Sepehrinezhad, Ali Shahbazi, Mohammad Taghi Joghataei, Fin Stolze Larsen, Sajad Sahab Negah

**Affiliations:** 1 Department of Neuroscience, Faculty of Advanced Technologies in Medicine, Iran University of Medical Sciences, Tehran, Iran; 2 Cellular and Molecular Research Center, Iran University of Medical Sciences, Tehran, Iran; 3 Department of Gastroenterology and Hepatology, Rigshospitalet, Copenhagen University Hospital, Copenhagen, Denmark; 4 Neuroscience Research Center, Mashhad University of Medical Sciences, Mashhad, Iran; 5 Department of Neuroscience, Faculty of Medicine, Mashhad University of Medical Sciences, Mashhad, Iran; 6 Shefa Neuroscience Research Center, Khatam Alanbia Hospital, Tehran, Iran

**Keywords:** Encephalopathy, Prognostic markers, Experimental models of disease, Acute inflammation, Hepatotoxicity

## Abstract

There is accumulating evidence that the circulatory levels of autotaxin (ATX) and lysophosphatidic acid (LPA) are increased in patients with severe liver disease. However, the potential role of the ATX-LPA axis in hepatic encephalopathy (HE) remains unclear. Our study aimed to investigate the role of the ATX-LPA signaling pathway in mice with thioacetamide (TAA) induced acute HE. To show the role of the ATX-LPA axis in the context of HE, we first measured the involvement of ATX-LPA in the pathogenesis of TAA-induced acute HE. Then, we compared the potential effects of ATX inhibitor (HA130) on astrocyte responses at in vitro and gut–liver–brain axis at in vivo levels. The inflammatory chemokine (C–C motif) ligand 3 was significantly increased in the hyperammonemic condition and could be prevented by ATX inhibition in astrocytes at in vitro level. Further statistical tests revealed that plasma and tissue pro-inflammatory cytokines were inhibited by HA130 in mice. Furthermore, the stage of HE was significantly improved by HA130. The most surprising result was that HA130 alleviated immune infiltrating cells in the liver and intestine and decreased mucus-secreting cells in the intestine. Further analysis showed that the levels of liver enzymes in serum were significantly decreased in response to ATX inhibition. Surprisingly, our data indicated that HA130 could recover permeabilization of the blood-brain barrier, neuroinflammation, and recognition memory. Besides that, we found that the changes of Interleukin-1 (IL-1) and aquaporin-4 (AQP4) in HE might have a connection with the glymphatic system based on bioinformatics analyses. Taken together, our data showed that the ATX-LPA axis contributes to the pathogenesis of HE and that inhibition of ATX improves HE.

## Introduction

Hepatic encephalopathy (HE) is a serious complication of advanced liver disease and acute liver failure that causes a variety of neurological consequences. The accumulation of gut-derived toxic substances (i.e., ammonia, short-chain fatty acids, benzodiazepine-like compounds, and mercaptans) and inflammation are considered the most relevant factors in the progression of the disease [1–3]. However, the pathophysiology of HE is multifactorial and the causes behind the development of cerebral dysfunctions following liver diseases are not yet fully elucidated. Overwhelming evidence suggests that the circulatory levels of autotaxin (ATX)-lysophosphatidic acid (LPA) are significantly increased in patients with liver-related disease [4–8]. ATX-LPA also seems to activate neuroinflammatory processes that result in the development of neurological complications [9–11]. The majority of LPA in the blood is synthesized from lysophosphatidylcholine through hydrolysis of its choline moiety by ATX [12]. LPA regulates various cellular signaling pathways and cellular processes, e.g., wound healing, differentiation, proliferation, migration, and survival via LPA G protein-coupled receptors, i.e., LPAR1-6 [13, 14]. Recently, the ATX-LPA axis was demonstrated to modulate the innate immune response in patients with acute liver failure via lysophosphatidic acid receptor 1 (LPAR1) and lysophosphatidic acid receptor 3 (LPAR3) [4]. However, the role of the ATX-LPA axis in the pathogenesis of HE is not clear. For this reason, the aim of the current study was to determine the role of the ATX-LPA axis during acute HE.

## Results

### Analyses of Astrocyte responses to the hyperammonemic condition and treatment with ATX inhibition

**Fig. 1:**
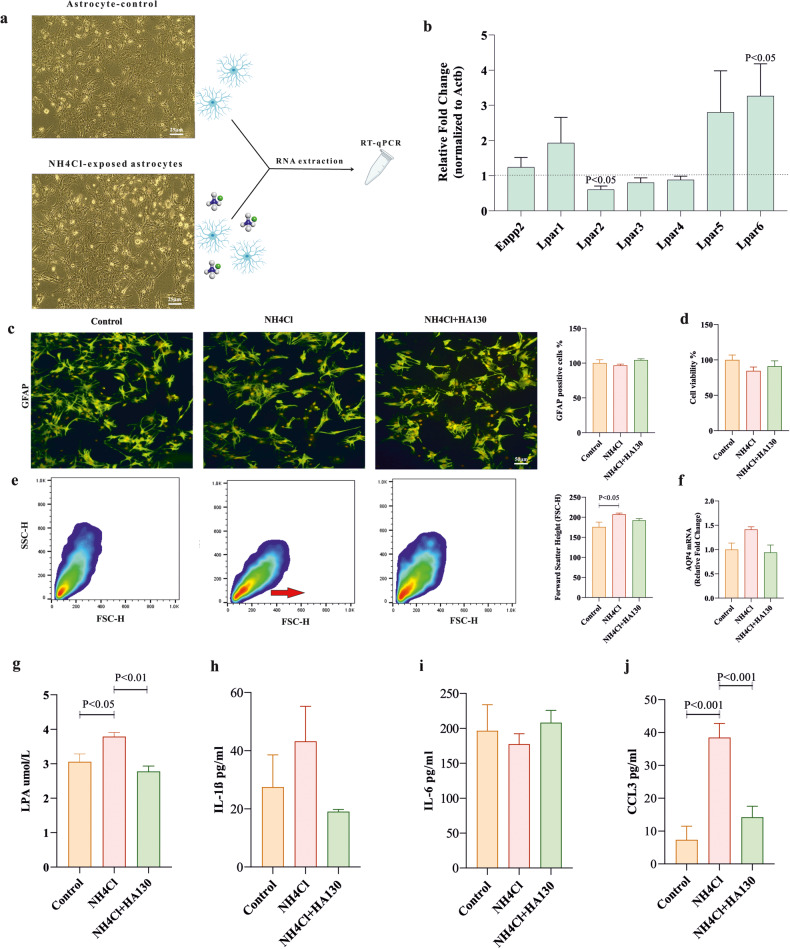
The responses of astrocytes in a hyperammonemic condition and treated by ATX inhibition. **a** Phase-contrast images of astrocytes in control and NH_4_Cl groups (*n* = 5/group) as well as a schematic diagram of the RT-PCR procedure are shown. **b** The expression of ectonucleotide pyrophosphatase/phosphodiesterase 2 (Enpp2; ATX gene) and lysophosphatidic acid receptors (LPARs) family at mRNA level in ammonia-exposed astrocytes. Fold-change for the control group is shown with a horizontal dotted line that equals one (*n* = 3/group). **c** Representative images of the expression of GFAP in different experimental groups are visualized by rabbit GFAP-specific antibody and anti-rabbit FITC antibody (green) using invert microscopy (*n* = 4/group). Cell nuclei were stained with PI (red). The bar graph indicated the percentage of GFAP-positive cells (*n* = 4/group). **d** The percentage of viable cells after exposure of the astrocyte cultures to NH_4_Cl and NH_4_Cl + HA130 using MTT assay (*n* = 5/group). **e** Flow cytometry measured forward scatter as an indicator of cell volume changes in different experimental groups. The bar graph represented the mean of forward scatter height (FSC-H) for all the experimental groups (*n* = 6–8/group). **f** AQP4 mRNA expression in ammonia-exposed astrocytes and treatment group were normalized to an internal reference (actin beta) compared to the control group (*n* = 3/group). **g**–**j** Sandwich ELISA method was used to measure the concentration of LPA, IL-1β, IL-6, and CCL3 in supernatants of astrocyte cultures after 24 h exposure with ammonia (5 mM) and ammonia-exposed astrocytes that were treated over 4 h with an autotaxin inhibitor (*n* = 3–5/group). Unpaired Student’s *t*-test test was used to compare fold-changes between control and ammonia groups in **b** and one-way ANOVA and post hoc Tukey’s multiple comparisons were performed to compare among all groups in **c**–**j**. Data are expressed as mean ± SEM. Aqp4 aquaporin-4, CCL3 chemokine (C–C motif) ligand 3, FSC-H forward scatter height, GFAP glial fibrillary acidic protein, IL-1β interleukin 1 beta, IL-6 interleukin 6, LPA lysophosphatidic acid, SSC-H side scatter height.

Our results indicated that NH_4_Cl significantly decreased the expression of lysophosphatidic acid receptor 2 (LPAR2) and increased the expression of lysophosphatidic acid receptor 6 (LPAR6) in astrocyte cultures at the mRNA level as compared to the control group (Fig. 1b; *P* < 0.05). To assess the astrocyte behavior in a hyperammonemic condition as seen in HE, astrocyte activation, proliferation, volume, and swelling were assessed. There were no significant differences in terms of activation as indicated by the expression of GFAP and proliferation as assessed by MTT in the ammonia-treated group compared to the control and HA130 groups (Fig. 1c, d). However, astrocyte volume was significantly increased in the ammonia-treated group compared to the control group assessed by flow cytometer (Fig. 1e; *P* < 0.05). Treatment of ammonia-exposed astrocytes with HA130 had no significant effect on cell volume (Fig. 1e). Finally, the expression of AQP4 water channels had no significant changes between different groups (Fig. 1f). To investigate the effects of hyperammonemic conditions and inhibition of ATX on LPA production, LPA concentration was measured using ELISA in supernatants samples. We found exposing astrocytes to NH_4_Cl had a significant increase in the level of LPA (Fig. 1g; *P* < 0.05). Interestingly, we observed a significant decrease in the levels of LPA after treating with HA130 during HA130 (Fig. 1g; *P* < 0.01). Furthermore, ammonia induced a significant increase in the production of CCL3 as inflammatory chemokine and ATX inhibition strongly suppressed ammonia-induced overproduction of CCL3 (Fig. 1j; *P* < 0.001) while the levels of IL-1β and IL-6 had no significant differences between the experimental groups (Fig. 1h, i).

### ATX inhibition attenuates severe infiltration of gut inflammatory cells and alleviates expression of colon goblet cells in TAA mice

**Fig. 2:**
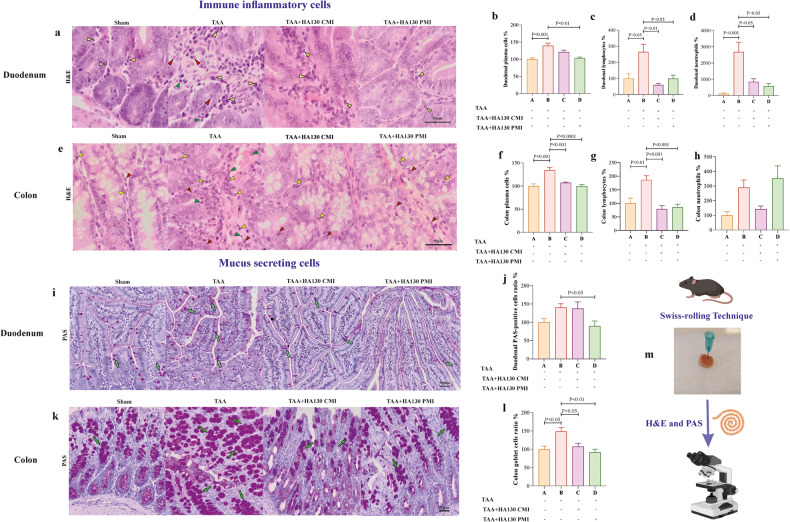
ATX inhibition decreases infiltration of immune cells and suppresses goblet cell proliferation into the gut in HE mice. **a** and **e** hematoxylin and eosin (H&E) stained sections in different groups represent the distribution of plasma cells (yellow arrowheads), lymphocytes (green arrowheads), and neutrophils (red arrowheads) in the lamina propria of duodenum and colon. Representative bar graphs present the plasma cells (**b** and **f**), lymphocytes (**c** and **g**), and neutrophils (**d** and **h**) in duodenal sections of experimental mice (*n* = 5–9/group). **i** and **k** PAS-stained duodenal sections display the number of goblet cells (green arrows) in duodenal and colon villi (*n* = 5–7/group). Representative bar graphs present the percentage of goblet cells in duodenal (**j**) and colon (**l**) sections of experimental mice (*n* = 5–9/group). **m** Schematic illustration shows the Swiss-role technique for microscopic examination of the intestine. Data are presented as mean ± SEM and analyzed one-way ANOVA and post hoc Tukey’s multiple comparisons. CMI concurrent model intervention, H&E hematoxylin and eosin, PAS periodic acid–Schiff, PMI post model intervention, TAA thioacetamide.

Analyzing the inflammatory duodenal cells showed HE could significantly increase the percentage of plasma cells, lymphocytes, and neutrophils compared to the sham group (Fig. 2a–d). We indicated that PMI and CMI intervention could dramatically inhibit the infiltration of lymphocytes into lamina propria in the duodenum and colon (Fig. 2c, g). Furthermore, the infiltration of neutrophils in the duodenum (Fig. 2d) and plasma cells in the colon (Fig. 2f) was significantly decreased in the treatment groups compared to the TAA group. We also found that mucus secretion was significantly improved in HE when treated by PMI intervention in the duodenum (Fig. 2c). Surprisingly, the mucus secretion indicated by goblet cells was improved in both treatment groups in the colon area.

### HE can affect the expression of the lysophosphatidic acid receptors family in liver and frontal cortex tissues

**Fig. 3:**
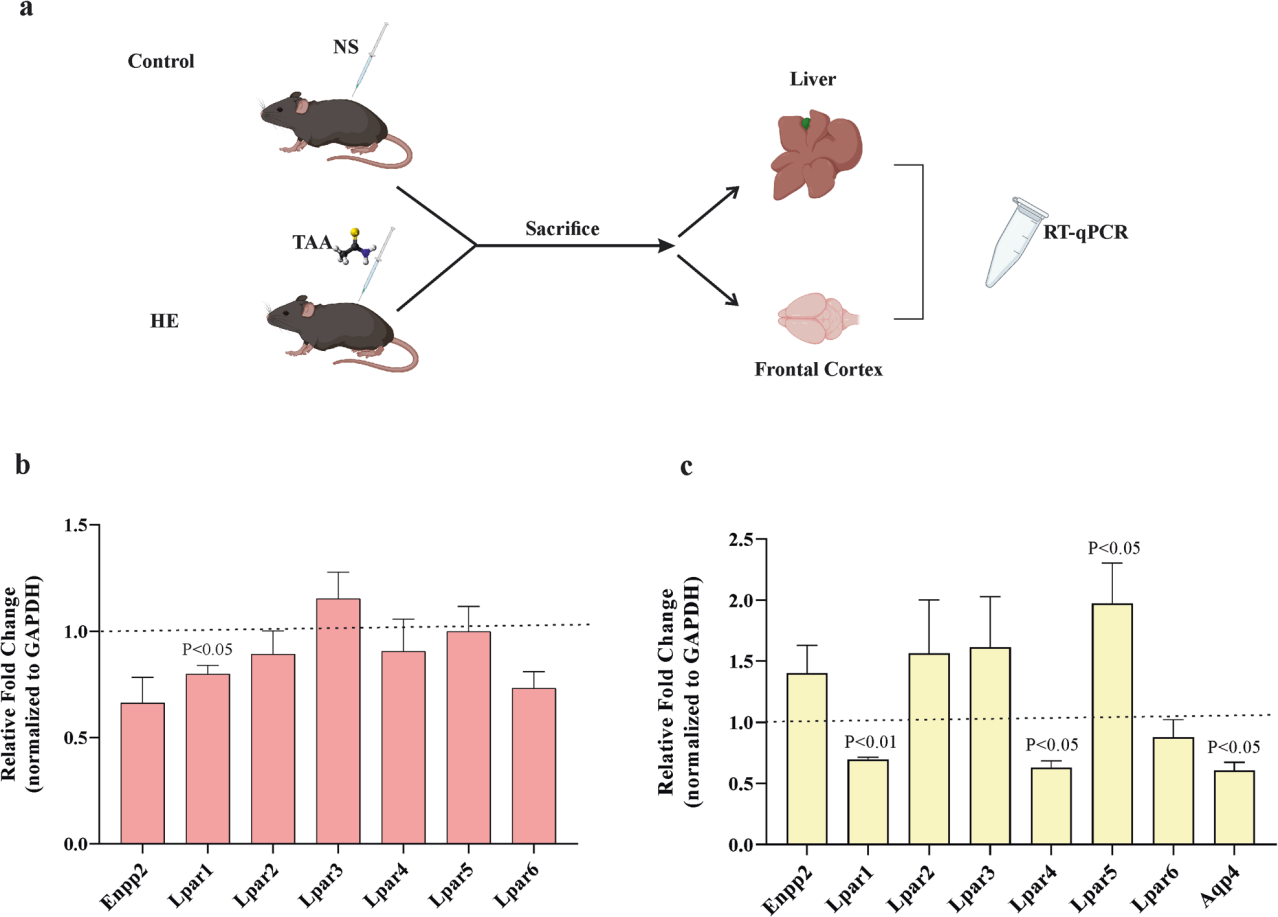
Hepatic encephalopathy differentially regulates the expression of LPARs in liver tissue and cerebral cortex of mice. **a** Schematic illustration of the experimental procedure. Samples were taken from target tissues and processed for mRNA expression. The mRNA expression of Enpp2 and LPARs family in liver (**b**) as well as AQP4 in cerebral cortex tissues (**c**) of HE mice, which were normalized to an internal reference (glyceraldehyde 3-phosphate dehydrogenase; GAPDH) compared to the control group (*n* = 4–5/group). Fold-change for the sham group is considered 1 as shown with horizontal dot lines. Data are expressed as mean ± SEM and analyzed by unpaired Student’s *t*-test test. HE hepatic encephalopathy, NS normal saline, RT-qPCR reverse transcription quantitative real-time polymerase chain reaction, TAA thioacetamide.

In the next set of experiments, we determined the gene expression level of lysophosphatidic acid receptors (LPARs) in liver tissue and frontal cortex of HE mice by reverse transcription quantitative real-time polymerase chain reaction (RT-qPCR) (Fig. 3a). Analyzing the results revealed that the expression of LPAR1 was significantly decreased in liver tissue of HE mice compared to the sham group (Fig. 3b). On the other hand, the mRNA expression of LPAR1, lysophosphatidic acid receptor 4 (LPAR4), and AQP4 were significantly decreased, while lysophosphatidic acid receptor 5 (LPAR5) was considerably increased in the cerebral cortex of HE mice compared to the sham group (Fig. 3c).

### ATX inhibition improves the stage of encephalopathy, leukocytosis, and thrombocytopenia in HE mice

The stage of encephalopathy is a main hallmark of HE; therefore, we compared this phenomenon to two types of interventions (i.e., CMI and PMI). We indicated that the stage of encephalopathy was dramatically decreased in treatment groups as compared to the control groups (Table 1; *P* < 0.001). In the next step, we compared hematological parameters in experimental groups to identify whether blood vital parameters can be affected by hyperammonemic encephalopathy with or without HA130 treatment (Table 1). The analysis revealed that the number of circulatory white blood cells significantly raised in the TAA group compared to the sham group, while the number of leukocytes significantly decreased when treated with HA130 in comparison with the TAA group (*P* < 0.01). Moreover, ATX inhibition could recover thrombocytopenia compared to the TAA group.

**Table 1:** Animal body weights, HE stages, and automated full blood analysis of experimental mice.

	Sham	TAA	TAA + HA130 CMI	TAA + HA130 PMI
*HE stage (I–IV) (n* *=* *7–11/group)*				
First day	0	2.3 ± 0.010 *(P* < 0.0001)^a^	2.57 ± 0.19	2.5 ± 0.21
Second day (2)	0	2.44 ± 0.11 *(P* < 0.0001)^a^	2	2.83 ± 0.15
Final day (3 or 5; 1 h before decapitation)	0	3.31 ± 0.15 (*P* < 0.0001)^a^	2 *(P* < 0.0001)^b^	2 (*P* < 0.0001)^b^
*Body weights (g) (n* *=* *13–19/group)*				
First day	27.90 ± 0.42	27.94 ± 0.24	27.63 ± 0.47	28.06 ± 0.55
Second day (2)	27.79 ± 0.45	26.43 ± 0.33	26.62 ± 0.49	27.94 ± 0.59
Final day (3 or 5; 1 h before decapitation)	27.87 ± 0.43	25.28 ± 0.35 *(P* < 0.001)^a^	24.88 ± 0.53	26.68 ± 0.54
*Hematology (n* *=* *5–7/group)*				
Leukocytes (×10^3^/mm^3^)	4.35 ± 0.51	6.7 ± 0.46 *(P* < 0.05)^a^	3.45 ± 0.22 *(P* < 0.01)^b^	3.58 ± 0.58 *(P* < 0.01)^b^
Red blood cells (×10^6^/mm^3^)	9.145 ± 0.13	10.542 ± 0.91	9.79 ± 0.53	9.37 ± 0.39
Hemoglobin (g/dl)	13.1 ± 0.26	15.54 ± 1.39	14.16 ± 0.82	13.57 ± 0.47
Hematocrit (%)	45.25 ± 0.84	50.92 ± 4.79	47.06 ± 2.73	45.74 ± 1.82
MCV (fl)	49.483 ± 0.41	48.14 ± 0.62	48.03 ± 0.38	48.85 ± 0.35
MCH (pg)	14.31 ± 0.20	14.72 ± 0.21	14.46 ± 0.18	14.52 ± 0.21
MCHC (g/dl)	28.78 ± 0.28	30.64 ± 0.45 *(P* < 0.01)^a^	30.11 ± 0.22	29.71 ± 0.31
RDW-CV (%)	14.95 ± 0.45	16.55 ± 0.71	18.08 ± 0.81	18.75 ± 0.96 *(P* < 0.05)^b^
RDW-SD (fl)	28.11 ± 0.19	27.88 ± 0.36	29.15 ± 0.15	30.31 ± 0.50 *(P* < 0.001)^b^
Platelets (×10^3^/mm^3^)	602 ± 133.4	96 ± 16.46 *(P* < 0.01)^a^	579.6 ± 110.78 *(P* < 0.05)^b^	696.85 ± 55.30 *(P* < 0.01)^b^
Serum albumin (mg/dl)	2.78 ± 0.10	2.75 ± 0.17	2.85 ± 0.09	3.16 ± 0.10

Data are represented as mean ± SEM and analyzed by one-way ANOVA and post hoc Tukey’s multiple comparisons.

*CMI* concurrent model intervention, *HE* hepatic encephalopathy, *MCH* mean corpuscular hemoglobin, *MCHC* mean corpuscular hemoglobin concentration, *MCV* mean corpuscular volume, *PMI* post model intervention, *TAA* Thioacetamide.

^a^Compared to Sham.

^b^Compared to TAA.

### ATX inhibition has excellent potential for alleviating the main hallmarks of HE

**Fig. 4:**
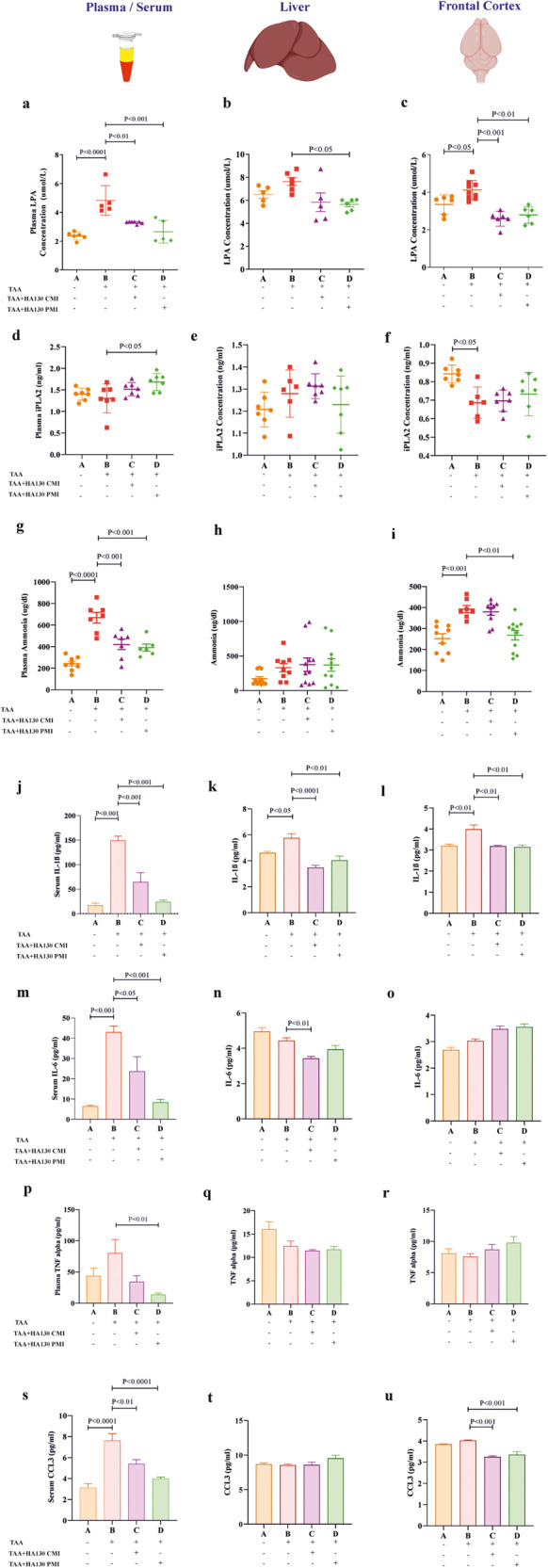
ATX inhibition attenuates HE pathogenesis in terms of LPA overproduction, hyperammonemic conditions, and inflammation in HE mice. The Sandwich ELISA method was used to measure the concentration of LPA (*n* = 5–9/group) and iPLA2 (*n* = 6*–*7/group) in plasma (**a** and **d**), liver (**b** and **e**), and frontal cortex (**c** and **f**) of experimental groups. The levels of ammonia in plasma (**g**), liver tissue (**h**), and frontal cortex (**i**) were assessed in different groups (*n* = 6–11/group). The concentration of pro-inflammatory cytokines IL-1β, IL-6, TNF alpha, and CCL3 in serum or plasma (**j**, **m**, **p**, **s**), liver tissue (**k**, **n**, **q**, **t**), and frontal cortex (**l**, **o**, **r**, **u**) in experimental groups (*n* = 5–9/group). Data are expressed as mean ± SEM and analyzed by one-way ANOVA and post hoc Tukey’s multiple comparisons. CMI concurrent model induction, CCL3 chemokine (C–C motif) ligand 3, CMI concurrent model intervention, IL-1β interleukin 1 beta, IL-6 interleukin 6, LPA lysophosphatidic acid, PMI post model intervention, TAA thioacetamide, TNF alpha tumor necrosis factor-alpha.

We found that the levels of LPA and ammonia were significantly increased in the plasma and frontal cortex of TAA-induced HE mice in comparison with the sham group (Fig. 4a, c). Our results indicated that HA130 (i.e., CMI and PMI) significantly inhibited the level of LPA in plasma and frontal cortex compared to the HE model (Fig. 4a, c). Furthermore, PMI intervention could suppress the level of LPA in the liver as compared to the TAA group (Fig. 4b; *P* < 0.05). Our results also show that HA130 increased the concentration of calcium-independent phospholipase A2 (iPLA2) in the circulation of PMI mice (Fig. 4d; *P* < 0.05). ELISA assay demonstrated the cerebral concentration of iPLA2 significantly decreased after HA130 administration in the PMI group (Fig. 4f). We found that the PMI approach could inhibit the level of ammonia in plasma and frontal cortex, while the inhibition of ammonia was just seen in plasma when treated by CMI compared to the TAA group (Fig. 4g, i). As revealed in the ELISA assay, the level of IL-1β was significantly increased in serum, liver, and frontal cortex, while the increased levels of IL-6 and CCL3 were seen in the serum of HE mice compared to the sham group (Fig. 4j–l, m, s). We next wanted to test whether ATX inhibition can modulate inflammatory responses. To this end, we treated HE mice with a potent ATX inhibitor. Injection of HA130 significantly inhibited the levels of IL-1β (in serum, liver, and frontal cortex), IL-6 (in serum), and CCL3 (in serum and frontal cortex) in intervention groups compared to the TAA group (Fig. 4j–l, m, s, u). In addition, CMI significantly decreased the concentration of IL-6 in liver tissues (Fig. 4n; *P* < 0.01), and PMI significantly reduced the levels of TNF alpha in plasma (Fig. 4p; *P* < 0.01) compared with the TAA group.

### ATX inhibition improved liver damage in HE

**Fig. 5:**
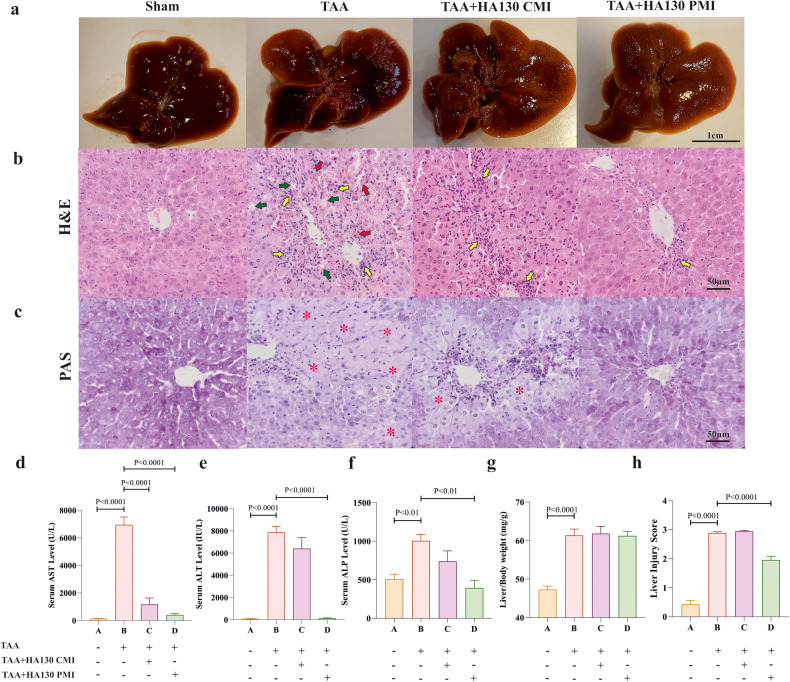
HA130 improved TAA-induced liver injury in mice. **a** Macroscopic view and morphological changes of the liver were seen in experimental groups. **b** Representative liver sections in H&E staining showed normal appearance in sham mice, leukocyte infiltration (yellow arrows), hepatocyte necrosis (green arrows), and severe hemorrhage (red arrows) in TAA group, only leukocyte infiltration in TAA + HA130 CMI, and almost normal appearance in TAA + HA130 PMI mice (*n* = 6–10/group). **c** Severe glycogen depletion in TAA mice in liver tissue was obvious, TAA + HA130 CMI mice showed moderate glycogen depletion, whileTAA+HA130 PMI mice displayed normal appearance in PAS staining liver sections (*n* = 5/group). The levels of serum-specific liver enzymes including AST (**d**), ALT (**e**), and ALP (**f**) in experimental groups (*n* = 6–7/group). **g** Bar graph of relative liver weights that were calculated as liver weights to body weights for experimental groups (*n* = 10–17/group). **h** H&E-stained-liver sections were quantitated by the severity of nuclear pyknosis, cytoplasmic hypereosinophilia, hepatocyte necrosis, hemorrhage, and neutrophil infiltration (*n* = 6–10/group). Data are expressed as mean ± SEM and analyzed by one-way ANOVA and post hoc Tukey’s multiple comparisons. ALP alkaline phosphatase, ALT alanine aminotransferase, AST aspartate aminotransferase, CMI concurrent model intervention, H&E hematoxylin and eosin, PAS periodic acid–Schiff, PMI post model intervention, TAA thioacetamide.

A variety of pathological changes in the liver was seen in the TAA model, as similarly seen in patients with HE. For instance, liver-specific enzymes (i.e., AST, ALT, and ALP), liver weight, severe necrosis, leukocyte infiltration, and glycogen shortage were significantly increased in the TAA model in comparison with the sham group (Fig. 5). We found that HA130 significantly reduced the level of AST in serum (Fig. 5d). In addition, serum levels of ALT and ALP were strongly decreased in PMI intervention compared to TAA group (Fig. 5e, f). Furthermore, observational examination of liver tissue indicated that some parameters including hepatocyte glycogen stores, leukocyte infiltration, hemorrhage, and hepatocyte necrosis were recovered when treated by HA130 in the PMI manner (Fig. 5b, c). Quantitative data from histological observation proved the efficacy of PMI intervention on pathological changes in the liver (Fig. 5h).

### ATX inhibition improved brain pathological changes after the TAA-induced HE model

**Fig. 6:**
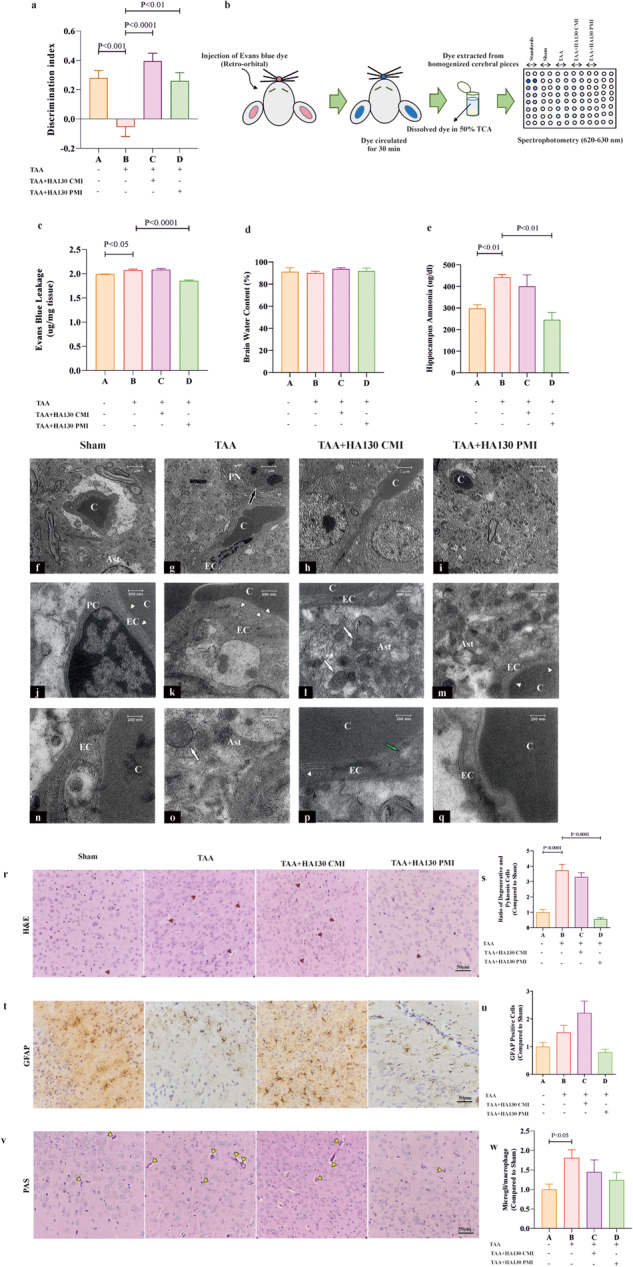
ATX inhibition improved HE-induced impaired recognition memory, recovered impaired blood-brain barrier permeability and improved cortical cells injury in HE mice. **a** Discrimination index as an indicator for learning and memory calculated based on novel object recognition test in experimental groups (*n* = 7–11/group). **b** Schematic illustration of Evans blue dye technique for assessing of the permeability of BBB in experimental groups is shown. **c** Bar graph indicates BBB permeability as quantified by Evans blue leakage in cortical pieces (*n* = 6–7/group). **d** The percentage of brain water content during the time course of the experiment (*n* = 6/group). **e** The levels of ammonia in the hippocampus of experimental groups (*n* = 4–5/group). Representative electron micrographs from the frontal cortex of saline-treated (**f**, **j**, **n**), TAA-treated (**g**, **k**, **o**), TAA + HA130 CMI treated (**h**, **l**, **p**) and TAA + HA130 PMI treated (**i**, **m**, **q**) mice. The black arrow in **g** points to a dark compacted pyramidal neuron, white arrowheads in **k** point to endothelial cytoplasmic projections and disrupted tight junctions, and the white arrow in **o** points to swelled mitochondria in an astrocyte cell in HE mice. White arrows in **l** point to swelled mitochondria in the astrocyte while green arrow and white arrowhead in **p** point to an enlarged mitochondrion and a disrupted tight junction of an activated endothelial cell in the TAA + HA130 CMI group. White arrowheads in **m** point to a normal-looking endothelial cell with intact tight junctions in TAA + HA130 PMI group. **r** H&E-stained brain sections showed pyknosis and degenerative cells (red arrowheads) in both TAA and TAA + HA130 CMI mice, while displaying normal structure in TAA + HA130 PMI group. **s** Bar graph has represented the ratio of degenerative cortical cells in experimental groups compared to the saline-treated control group (*n* = 6–10/group). **t** Immunohistochemical staining against GFAP as an astrocyte marker was performed. **u** Bar graph indicates the ratio of GFAP-positive cells (brown cells) in the frontal cortex in different experimental groups (*n* = 5/group). **v** PAS staining sections show activated microglia/macrophages in the frontal cortex of different experimental groups. **w** Bar graph represents the ratio of PAS-positive cells (yellow arrowheads) in the cerebral cortex (*n* = 7–9). Data are expressed as mean ± SEM and analyzed by one-way ANOVA and post hoc Tukey’s multiple comparisons. Ast astastrocyte, C capillary, CMI concurrent model intervention, EC endothelial cell, GFAP glial fibrillary acidic protein, H&E hematoxylin and eosin, PC pericyte, PMI post model intervention, PN pyramidal neuron, TAA thioacetamide, TCA trichloroacetic acid.

To show the effects of ATX inhibition on TAA-induced HE, several variables, such as BBB permeability, brain water content, level of ammonia, and novel objective tests were assessed. Our data indicated that administration of HA130 robustly improved recognition memory compared to the TAA group (Fig. 6a). Moreover, the permeabilization of BBB assessed by Evans blue method was significantly recovered in the frontal cortex of TAA + HA130 PMI mice (Fig. 6c). Results of brain water content indicated no significant differences between experimental groups (Fig. 6d). To identify whether TAA and ATX inhibition can affect ammonia levels in the hippocampus, we used an ammonia assay kit. The results of ammonia levels in the hippocampus showed that HA130 significantly suppressed hyperammonemic conditions in the hippocampus of TAA mice (Fig. 6e). To confirm the sealing of BBB, ultrastructure examination was performed by transmission electron microscopy (TEM). Disrupted tight junctions along with cytoplasmic projections of endothelial cells and swollen mitochondria in peri-vascular astrocytes were obvious in the HE group by evaluating TEM images (Fig. 6g, k, o). Interestingly, an improvement was seen in the ultrastructure indicators (i.e., tight junctions and mitochondria) in PMI intervention (Fig. 6I, m, q). Histopathological examination indicated that the ratio of pyknotic cells was significantly increased in HE mice, while PMI intervention could inhibit this indicator in the frontal cortex compared to the TAA group (Fig. 6r, s). Furthermore, the astrocytes activation marked by GFAP was evaluated and there were not any significant changes at this level between groups (Fig. 6t, u). Also, analyzing PAS-stained cerebral cortex sections showed a significant increase in the number of activated microglia/macrophages in HE mice compared to the sham group (Fig. 6v, w).

### Reconstructed genetic networks and their relationship to IL-1β

**Fig. 7:**
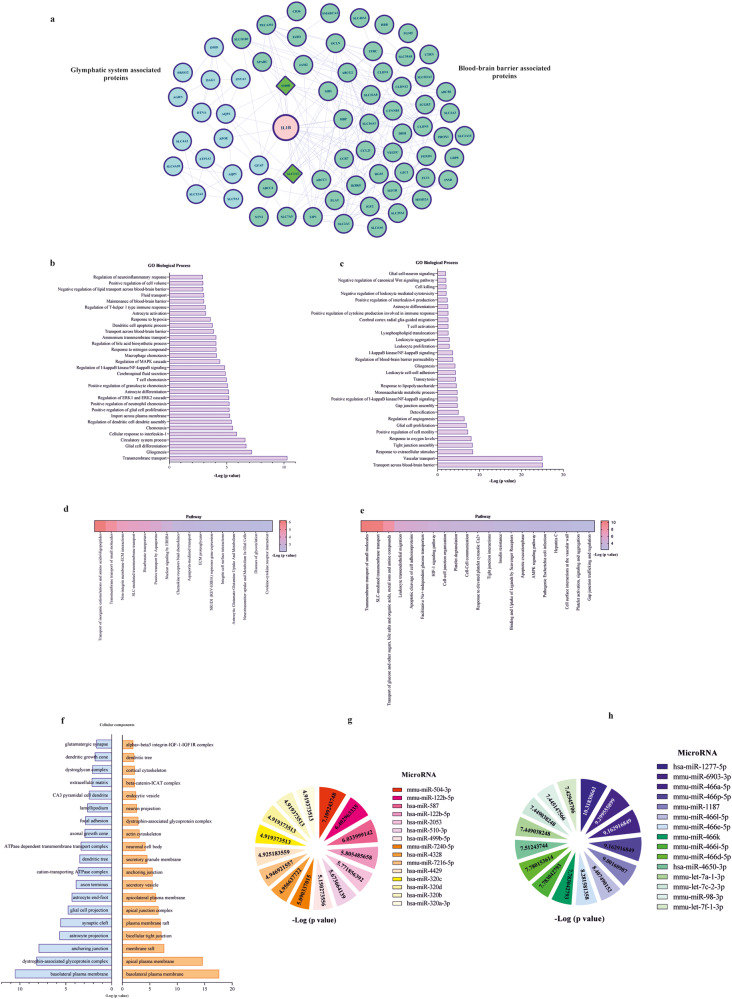
Genetic network, GO analysis, enriched Reactome pathway, and microRNAs prediction of both glymphatic system and the BBB associated genes and IL-1β. **a** Reconstructed genetic network of genes associated with the glymphatic system (blue nodes), BBB-related genes (green nodes), and IL-1β. Diamond-shaped nodes are common between the two networks. **b** and **c** GO biological enrichment analysis of genes associated with glymphatic system+IL-1β (**b**) and gene related to BBB + IL-1β (**c**). **d** and **e** Reactome pathway analysis for IL-1β along with genes related to the glymphatic system (**d**) and BBB (**e**). **f** Cellular component enrichment analysis of IL-1β along with genes related to the glymphatic system (blue plot) and BBB (orange plot). **g** Predicted microRNA for genes associated with the glymphatic system and IL-1β. **h** Enriched microRNA for genes associated with the BBB and IL-1β.

After conducting a review of existing literature, we identified a total of 23 genes associated with the glymphatic system and 55 genes associated with the BBB structure (Supplementary Tables 1 and 2). Upon reconstructing the genetic networks for each set of genes, we found that 6 nodes in the glymphatic system and 2 nodes in the BBB network were not connected to any other nodes. We then evaluated the potential interactions between each genetic network and IL-1β, and identified two genes (S100β and SLC1A3) that were shared between the glymphatic system and BBB networks. In the genetic networks for the glymphatic system, 5 genes were connected to IL-1β, while 18 genes were connected to IL-1β in the BBB network (Fig. 7a).

### Gene ontology and Reactome pathway enrichment analysis

Gene ontology and Reactome pathway enrichment analysis revealed the biological processes and pathways that may play a role in glymphatic and BBB impairment by IL-1β (Fig. 7). For glymphatic impairment, GO analysis showed that gliogenesis, cellular response to interleukin-1, macrophage chemotaxis, MAPK-ERK1/2 cascade, ammonium transport, and neuroinflammatory response were main biological processes (Fig. 7b). Reactome pathway analysis predicted nuclear signaling by ENBB4, chemokine receptors binding chemokines, aquaporin-mediated transport, neurotransmitter uptake and metabolism in glial cells, and cytokine-cytokine receptor interaction as the main involved pathways (Fig. 7d). The most affected cellular components that may cause glymphatic system impairment by IL-1β were the dystrophin-associated glycoprotein complex, glial cell projection, astrocyte end-foot, and cation-transporting ATPase complex (Fig. 7f blue chart). For BBB disruption, GO analysis showed that glial cell proliferation, I-kappaB kinase/NF-kappaB signaling, response to lipopolysaccharide, leukocyte cell-cell adhesion, gliogenesis, leukocyte aggregation, lysophospholipids translocation, T-cell activation, IL-6 production, and canonical Wnt signaling may mediate BBB damage by IL-1β (Fig. 7c). Reactome pathway analysis predicted leukocyte transendothelial migration, apoptotic cleavage of cell adhesion proteins, HIF-1 signaling pathway, apoptotic execution phase, and AMPK signaling pathway as the main involved pathways (Fig. 7e). The most affected cellular components that may cause BBB damage by IL-1β were the apical plasma membrane, bicellular tight junction, secretory vesicle, anchoring junction, endocytic vesicle, and cortical cytoskeleton (Fig. 7f orange chart).

### MicroRNA prediction

MicroRNA target prediction identified several significant microRNAs for genes associated with the glymphatic system and BBB (Fig. 7g, h). For genes associated with the glymphatic system and IL-1β, the most significant microRNAs were mmu-miR-504-3p, mmu-miR-122b-5p, and hsa-miR-587, with mmu-miR-504-3p and hsa-miR-587 targeted to 6 genes and mmu-miR-122b-5p targeted to 5 genes (Supplementary Table 3). For BBB and IL-1β predicted microRNAs, the most significant microRNAs were hsa-miR-1277-5p, mmu-miR-6903-3p, mmu-miR-466a-5p, mmu-miR-466p-5p, and mmu-miR-1187, with hsa-miR-1277-5p predicted to 15 genes and mmu-miR-6903-3p targeted to 12 genes, while mmu-miR-466a-5p, mmu-miR-466p-5p, and mmu-miR-1187 were targeted to 9 genes (Supplementary Table 4).

## Discussion

**Fig. 8:**
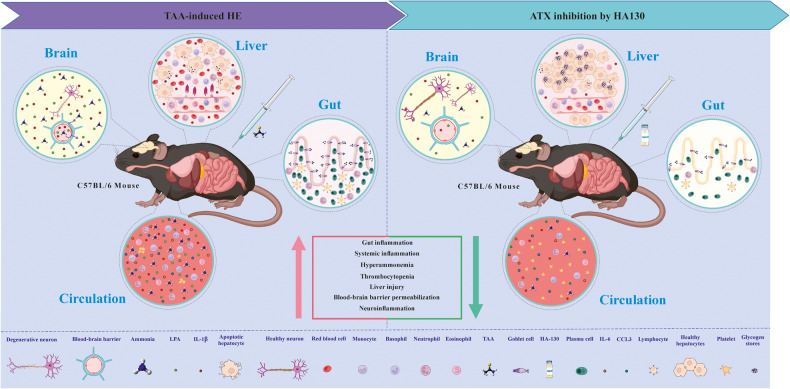
Summary of the in vivo effects of HA130 on gut–liver–brain axis in the experimental mice model of HE. Two consecutive intraperitoneal injections of thioacetamide produced intestinal inflammation, systemic inflammation, liver injury, hyperammonemia and neuroinflammation in mice. Inhibition of autotaxin by HA130 significantly alleviated these pathological conditions in HE mice.

We hypothesized that inhibition of ATX by the selective inhibitor HA130 would alleviate the gut–liver–brain axis and improve HE. First, we confirmed the involvement of the ATX-LPA signaling axis in the development of HE (Fig. 8). Then, we evaluated the therapeutic potential effects of HA130 in TAA-induced acute liver injury and found that hyperammonemia altered some of the mRNA expressions of LPARs in astrocytes (i.e., LPAR2 and 6) together with an increase in the astrocyte volume. HA130 also significantly decreased the levels of LPA and CCL3 in astrocytes exposed to hyperammonemic conditions. Our results indicated that the expression of LPARs in the context of HE is so complex and depends on the target tissue. For instance, some receptors, such as LPAR1 and 4 were suppressed, while LPAR5 was significantly increased after HE in the cerebral cortex. The stage of encephalopathy as the main hallmark of HE was decreased in treatment groups as compared to the control groups. Taken together, our findings demonstrated that hyperammonemic conditions and a high amount of LPA were significantly improved at the histological and serological levels in the treatment groups compared to the HE group.

The intestinal tract is a major source of ammonia in liver failure. Gut dysbiosis is associated with central nervous system (CNS) dysfunction in terms of brain histopathology and substance metabolism. For example, histopathological changes, such as brain microvessel endothelial cell damage, microglial activation, astrocyte swelling, and neuronal damage were taken place after gut dysbiosis. Moreover, the concentration of gamma-aminobutyric acid and the expression of serotonin receptors were significantly altered after gut dysbiosis [15]. Evaluating the structure of the intestinal tract can promote the management of HE. We compared immune infiltrating cells and mucus-secreting cells and found that HA130 could recover the structure and function of the intestinal tract. Overall, we found that inflammatory cytokines were lower in the treatment groups. Surprisingly, CNS-related parameters including cognitive impairment, BBB disruption at the ultrastructural analysis, and cellular damage were profoundly relieved in the treatment group, especially PMI intervention.

The significant upregulation of the ATX-LPA signaling axis has been detected in humans with different liver diseases [4, 5, 16]. The increased serum level of ATX is induced by a lower capacity of the liver sinusoidal endothelial cells, which causes deterioration of liver function by producing cytokines and recruiting inflammatory cells [17]. In our study, we indicated that inflammatory cytokines (i.e., IL-1β, IL-6, and CCL-3) were profoundly inhibited by ATX inhibition. We also demonstrated that recruiting inflammatory cells in the gut and liver was correlated with an increase in HE, and HA130 alleviates the expression of immune infiltrating cells in those organs. In addition to the inflammatory background, we measured the ammonia level and liver function markers (i.e., AST, ALT, ALP, liver/body weight) in response to ATX inhibition. Our results revealed that thrombocytopenia and ammonia levels were recovered by HA130 in PMI intervention. This outcome is contrary to that of Roy et al. who found treatment with the autotaxin inhibitor PF-8380 did not affect liver function markers including AST, ALT, and total bilirubin and no change in ammonia levels in the brain and plasma [11]. By comparing the studies, the HE model was similar between studies; however, the type of ATX inhibitor is different. It can be suggested that the type of ATX inhibitor may have a critical role in downstream pathways. Furthermore, the time of action of the ATX inhibitor can affect the outcomes. For instance, we observed that when HA130 was used at the chronic phase (PMI) had better results than that of an early stage of HE (CMI intervention).

Another important consequence of HE is brain-related injuries. For example, the high level of LPA seems to have a central role in increasing BBB permeability [18], which we here demonstrated that the permeability was mitigated by ATX inhibition. In accordance with the present results, a recent study has demonstrated that inhibition of ATX improves neurological dysfunction in acute HE by reducing neuroinflammatory cytokines [11]. Microglia are the resident immune cells in the brain and play a critical role in the brain’s immune response. They can become activated in response to injury or infection and release inflammatory mediators, which can lead to neuroinflammation and neuronal damage [19–21]. Studies have shown that microglia activation occurs in the brain during HE and that this activation is associated with cognitive dysfunction and neuroinflammation [22, 23]. In addition, the activation of microglia may contribute to the development and progression of HE by releasing pro-inflammatory cytokines and exacerbating the inflammatory response [24–26]. Furthermore, recent research has suggested that the gut–brain axis may play a role in the development of HE, as gut-derived products such as bacterial endotoxins can activate microglia and contribute to neuroinflammation. However, further research is needed to fully understand the relationship between microglia activation and hepatic encephalopathy. Our results suggest that microglia activation may contribute to the development and progression of this condition.

There is some evidence to suggest a potential link between the expression of aquaporin-4 (AQP4) and hepatic encephalopathy (HE) through the glymphatic system [27–29], although the exact nature of this relationship is still being studied. Several studies have investigated the expression of AQP4 in the context of HE [30, 31]. One study found that AQP4 expression was significantly increased in the brains of rats with acute liver failure, which is a common cause of HE [32]. Another study found that the expression of AQP4 was dramatically decreased in the cortex, hippocampus, and midbrain of rats with HE, and treatment with lactulose, a commonly used therapy for HE, was tended to increase AQP4 expression [31]. In the current study, we found that the expression of AQP4 was significantly decreased at the mRNA level in an acute HE model. In accordance with previous studies, our findings suggest that AQP4 may be involved in the pathogenesis of HE through the glymphatic system. However, more research is needed to fully understand the relationship between AQP4 expression and HE through the glymphatic system. For this reason, we did a bioinformatics analysis to predict the downstream of IL-1β in connection with the glymphatic system and BBB disruption. Our data showed that neuroinflammatory responses and glial cell-neuron signaling might play vital roles in the progression of HE through different miRNAs and cellular components related to the glymphatic system failure. These findings encourage us to investigate the underlying mechanisms and potential therapeutic targets for this complex disorder.

This study is limited by the lack of information on the ATX activity in plasma, liver tissue, and frontal cortex of experimental mice. Another issue that was not addressed in this study was the expression of calcium-binding adapter molecule 1 (Iba1) as a specific marker for microglia, which could have given us more insights into possible phenotypes of these inflammatory cells in the brain parenchyma. However, we evaluated the PAS-positive cells as an indication of microglia/macrophages in brain slices and found that PAS-positive cells were significantly increased in the HE group, indicating the increased activity of microglia/macrophages during HE. We also found that the expression of AQP4 was significantly changed in HE model, it might be due to the impairment in the glymphatic system; however, it should be investigated carefully in future studies. Another limitation of our study is that we did not perform LPAR antagonism experiments to investigate the functional significance of LPAR expression in the hyperammonia conditions. Although we measured LPAR expression levels by RT-PCR, the specific signaling pathways and physiological functions of different LPAR subtypes may vary. Future investigations could include LPAR antagonism experiments targeting LPAR1, 4, or 5 to further elucidate the mechanistic relevance of LPA and its receptors in hyperammonia-induced astrocyte dysfunction.

In conclusion, our data suggest that ATX-LPA signaling contributed to the pathophysiology of HE by promoting the generation of pro-inflammatory cytokines and chemokines. The beneficial effects of HA130 could be mediated through ammonia scavenging and anti-inflammatory effects, which alleviates systemic inflammation, gut leukocyte infiltration, liver inflammation, neuroinflammation, hyperammonemia, and cerebral ammonia level. To predict possible downstream changes followed by HE, we did a bioinformatics analysis and indicated that there are several genetic and cellular connections between HE and the glymphatic system. Further studies are needed to figure out the molecular underpinnings. Furthermore, targeting the ATX-LPA axis could be a new and interesting intervention to prevent HE but requires further study with a special focus on the efficacy of each LPARs antagonists.

## Methods

### Ethical statement

All procedures and experimental approaches were performed and reported according to the Animal Research: Reporting of In Vivo Experiments (ARRIVE) guidelines [33] and were approved by the Animal Care and Use Committee (ACUC) at the Iran University of Medical Sciences under the ethical code of IR.IUMS.REC.1398.959, which was established by the National Institutes of Health guidelines [34].

### Astrocyte cultures

To find out the role of ATX on astrocytes’ behavior, such as proliferation, swelling, and activation, astrocytes derived from mouse pups were cultured and treated with ammonium chloride (NH_4_Cl) as an in vitro model for HE, and HA130 as a potent inhibitor for ATX. Primary cultures of astrocytes of neonate mice cerebral cortex (postnatal day 2–5; P2–P5) were prepared as previously described [35]. C57BL/6 mouse pups were purchased from the animal house of Iran University of Medical Sciences. The P2–P5 mice were decapitated after sedation on −20 °C ice pads. After removal of the meninges, some pieces of the cerebral cortex were exited and manually homogenized with a scalpel in phosphate buffer saline (PBS; containing 137 mM NaCl, 2.7 mM KCl, 10 mM Na_2_HPO_4_, 1.8 mM KH_2_PO_4_, pH ~7.4) for 3 min at room temperature. Then, homogenized tissues were obtained by centrifugation at 1500 rpm for 5 min. Tissue plates were digested with 0.25% trypsin solution (Sigma-Aldrich, Germany) for 3 min. After that, cell plates were obtained by centrifugation and cultured in DMEM/F12 medium (Gibco Thermo Fisher Scientific, UK) containing 20% fetal bovine serum (FBS; Gibco Thermo Fisher Scientific, UK), 1% penicillin-streptomycin (Sigma-Aldrich, Germany), and 1% amphotericin B solution (Sigma-Aldrich, Germany) as the completed medium. Culture cells were incubated at 37 °C with 5% CO_2_ and 95% air. The culture medium was changed every 2–3 days. When 70-80% of the surface of the culture vessel is filled by mixed glial cells, astrocyte purification was performed by the shaking method. First of all, culture vessels were shaken at 180 rpm for 30 min by a 37 °C shaker incubator (SCI FINETECH Co, Korea) for eliminating microglia cells. Next, supernatants were discarded and culture flasks were washed twice with a PBS solution containing 1% penicillin-streptomycin. Then, the completed medium was added to the culture flasks and shaken again at 240 rpm for 6 h to eliminate oligodendrocyte precursor cells. Later on, supernatants were discarded, and pure astrocytes were detached from the culture flask and re-culture in a new culture vessel and were maintained until day 15 after cultivation. On day 15 post-seeding the 20% FBS was replaced with 10% FBS to induce maturation of astrocytes. To characterize astrocytes, isolated cells were incubated against the glial fibrillary acidic protein (GFAP) using the immunocytochemistry method. All experiments were conducted on days 25–28 post-seeding.

### Treatment of cultures

To show the effects of ATX inhibitor on astrocytes behavior, three different experimental conditions including control, NH_4_Cl, and NH_4_Cl + HA130 were prepared. Astrocytes in the control group had completed medium. Ammonia groups were incubated with 5 mM ammonium chloride (TITRACHEM, Iran; diluted in sterile water) for 24 h [36]. After incubation of astrocytes with ammonia, cells were also incubated with a potent autotaxin inhibitor (HA130; 20 µM; Cayman Chemical, USA) for 4 h in the third group [37]. The details of in vitro experiment are provided in the Supplementary Methods.

### Thioacetamide mouse model of HE

Ten-to-twelve-week-old male C57BL/6 mice (25–30 g; 122 mice) were obtained from the animal house of Iran University of Medical Sciences. Animals were maintained in a 12–12 h light/dark cycle room and were allowed free access to food (rodent chow) and water (in bottle) in a condition with 50–60% humidity and 24 ± 1 °C. Acute liver failure induced-HE was induced by 2–3 consecutive injections of thioacetamide (TAA; Sigma-Aldrich, USA) [24, 38]. Briefly, TAA (600 mg/kg) was dissolved in 150 µL normal saline and was injected intraperitoneally. Afterward, TAA was injected 24 h after injection for the second time. All animals were free to access food and wet pellets on the cage floor. A 250 µL maintenance solution containing 5% dextrose in normal saline was daily injected subcutaneously to prevent hypoglycemia and dehydration. Animal weights were also daily recorded and mice that lost more than 20% of their weight were excluded from the experiment.

### Experimental groups and therapeutic interventions

After adaptation of all animals to the environment, the mice were randomly divided into four groups (not blinded): A as sham-control groups, which received 150 µL normal saline intraperitoneally; B as TAA groups, which were injected 2 consecutive dosages of TAA during 48 h; C as TAA + HA130 concurrent model intervention (CMI) groups, that were injected 2 consecutive dosages of TAA and were treated with six dosages of a potent autotaxin inhibitor HA130 At the same time with induction of model by TAA; D as TAA + HA130 post model intervention (PMI), that were injected 2 consecutive dosages of TAA and were treated with six dosages of HA130 after induction of model by TAA. HA130 at a dose of 463 µg/kg was dissolved in DMSO and then diluted with 100 µL normal saline and injected intraperitoneally in treatment groups. As previously described, this dose of HA130 strongly decreased plasma levels of LPA in mice [39]. Six dosages of HA130 (twice a day for 3 days) were injected to inhibit permanently the plasma level of LPA and keep its concentration low (Supplementary Fig. 1). The details of in vivo experiment are provided in the Supplementary Methods.

### Quantitative and statistical analyses

All statistical analyses were performed using GraphPad Prism version 8.3.0. Data are presented as mean ± standard error of the mean (SEM). The unpaired Student’s *t*-test was used to compare two experimental groups. Furthermore, one-way ANOVA followed by Tukey’s post hoc tests was performed to comparison between more than two experimental groups. In all statistical analyses, a *P*-value less than 0.05 was considered statistically significant.

### Bioinformatic analysis

We used a bioinformatics approach to predict the possible signaling pathways affected by IL-1β that contribute to the disruption of the BBB and impairment of the glymphatic system. We first collected all genes associated with the glymphatic system and BBB from the literature. The genes were then uploaded into STRING to visualize the protein-protein interactions, and Cytoscape was used to create genetic networks for both the glymphatic system and BBB. We then performed GO (Gene ontology) enrichment analysis to identify the main biological processes and cellular components involved, and Reactome was used to specify the signaling pathways. To predict the microRNAs associated with each gene set and IL-1β, we used miRDB. All analyses were performed using the ToppFun section of ToppGene (https://toppgene.cchmc.org/) with a cutoff value of *P* < 0.05 [40]. Finally, the results were presented in heat map charts and bar plots generated using GraphPad Prism software.

## Supplementary information

Supplementary File

Supplementary Figure 1

aj-checklist

## Data Availability

All relevant data supporting the key findings of this study are available within the article and its Supplementary Information files or from the corresponding author upon reasonable request.
