# Supplementary material for: Inhibition of autotaxin alleviates pathological features of hepatic encephalopathy at the level of gut–liver–brain axis: an experimental and bioinformatic study
Source: Cell Death Dis. 2023 Aug 1;14(8):490. doi: 10.1038/s41419-023-06022-5 (PMC10394058; doi:10.1038/s41419-023-06022-5)
Supplement: Supplementary file 1 — Supplementary File [file 41419_2023_6022_MOESM1_ESM.docx]

**Inhibition of autotaxin alleviates pathological features of hepatic encephalopathy at the level of gut-liver-brain axis: an experimental and bioinformatic study**

**Table of Contents**

[**Supplementary Results** 2](#_Toc138690368)

[Supplementary Table 1. Gene associated to glymphatic system. 2](#_Toc138690369)

[Supplementary Table 2. Gene associated to the blood-brain barrier. 3](#_Toc138690370)

[Supplementary Table 3. Predicted miRNAs for gene associated to glymphatic system and IL-1β. 4](#_Toc138690371)

[Supplementary Table 4. Predicted miRNAs for gene associated to the blood-brain barrier and IL-1β. 5](#_Toc138690372)

[**Supplementary Methods** 6](#_Toc138690373)

[Supplementary Fig 1. Schematic illustration of the protocol used for in vitro, in vivo, and bioinformatic experiments in the study. 6](#_Toc138690374)

[Immunofluorescence of GFAP in astrocyte cultures 6](#_Toc138690375)

[Cell viability assay 6](#_Toc138690376)

[Flow cytometry and determination of cell volume changes 7](#_Toc138690377)

[Novel object recognition test and clinical stage of encephalopathy 7](#_Toc138690378)

[Hematological analysis 7](#_Toc138690379)

[Enzyme-linked immunosorbent assays 8](#_Toc138690380)

[Measurement of ammonia level 8](#_Toc138690381)

[Liver function tests and relative tissue weight 8](#_Toc138690382)

[Cerebral edema 9](#_Toc138690383)

[Tissue preparation for histopathology and immunohistochemistry 9](#_Toc138690384)

[Hematoxylin and eosin staining for histopathology 9](#_Toc138690385)

[Periodic acid‑Schiff staining for intestines, liver and cerebral cortex samples 10](#_Toc138690386)

[Immunohistochemistry 10](#_Toc138690387)

[Blood-brain barrier permeability 11](#_Toc138690388)

[Ultra-structural examination of the blood-brain barrier 11](#_Toc138690389)

[Ribonucleic acid extraction, complementary deoxyribonucleic acid synthesis and quantitative reverse transcriptase real-time polymerase chain reaction 11](#_Toc138690390)

[Supplementary Table.5 Real-time PCR primer information. 12](#_Toc138690391)

[Supplementary Table.6 List of used antibodies 13](#_Toc138690392)

[Supplementary Table.7 List of used organisms 13](#_Toc138690393)

[Supplementary Table.8 List of used software 13](#_Toc138690394)

[Supplementary Table.9 List of used agent/drug 14](#_Toc138690395)

[**Supplementary References** 14](#_Toc138690396)

# **Supplementary Results**

| Supplementary Table 1. Gene associated to glymphatic system. | | | |
| --- | --- | --- | --- |
| Index | **Gene symbol** | **Full name** | **Ensembl ID** |
|  | AQP4 | Aquaporin 4 | ENSG00000171885 |
|  | Nr1d1 | Nuclear receptor subfamily 1 group D member 1 | ENSG00000126368 |
|  | DTNA | Dystrobrevin alpha | ENSG00000134769 |
|  | L1cam | L1 cell adhesion molecule | ENSG00000198910 |
|  | DAG1 | Dystroglycan | ENSG00000173402 |
|  | SNTA1 | Syntrophin | [ENSG00000101400](http://www.ensembl.org/id/ENSG00000101400) |
|  | DMD | Dystrophin | [ENSG00000198947](http://www.ensembl.org/id/ENSG00000198947) |
|  | AQP1 | Aquaporin 1 | ENSG00000240583 |
|  | GFAP | Glial fibrillary acidic protein | [ENSG00000131095](http://www.ensembl.org/id/ENSG00000131095) |
|  | PROX-1 | Prospero homeobox 1 | [ENSG00000117707](http://www.ensembl.org/id/ENSG00000117707) |
|  | APOE | Apolipoprotein E | [ENSG00000130203](http://www.ensembl.org/id/ENSG00000130203) |
|  | VEGFC | Vascular endothelial growth factor-C | ENSG00000150630 |
|  | CCL21 | C-C motif chemokine ligand 21 | [ENSG00000137077](http://www.ensembl.org/id/ENSG00000137077) |
|  | CCR7 | C-C chemokine receptor type 7 | [ENSG00000126353](http://www.ensembl.org/id/ENSG00000126353) |
|  | EAAT1 | Excitatory amino acid transporter 1 | [ENSG00000079215](http://www.ensembl.org/id/ENSG00000079215) |
|  | S100B | S100 calcium binding protein B | [ENSG00000160307](http://www.ensembl.org/id/ENSG00000160307) |
|  | AGRN | Agrin | [ENSG00000188157](http://www.ensembl.org/id/ENSG00000188157) |
|  | PRSS12 | Neurotrypsin | [ENSG00000164099](http://www.ensembl.org/id/ENSG00000164099) |
|  | ATP1A1 | ATPase Na^+^/K^+^ transporting subunit alpha 1 | [ENSG00000163399](http://www.ensembl.org/id/ENSG00000163399) |
|  | Slc4a10 | Solute carrier family 4 member 10 | ENSG00000144290 |
|  | SLC9A1 | Solute carrier family 9 member A1 | [ENSG00000090020](http://www.ensembl.org/id/ENSG00000090020) |
|  | SLC12A2 | Solute carrier family 12 member 2 | ENSG00000064651 |
|  | SLC4A2 | Solute carrier family 4 member 2 | [ENSG00000164889](http://www.ensembl.org/id/ENSG00000164889) |

| Supplementary Table 2. Gene associated to the blood-brain barrier. | | | |
| --- | --- | --- | --- |
| Index | **Gene symbol** | **Full name** | **Ensembl ID** |
|  | PLAT | Plasminogen activator, tissue type | ENSG00000104368 |
|  | IGF2 | Insulin like growth factor 2 | ENSG00000167244 |
|  | FLT1 | fms related receptor tyrosine kinase 1 | ENSG00000102755 |
|  | BTG2 | BTG anti-proliferation factor 2(PC3) | ENSG00000159388 |
|  | RGS5 | Regulator of G protein signaling 5 | ENSG00000143248 |
|  | UTRN | Utrophin | ENSG00000152818 |
|  | IKBKE | Inhibitor of nuclear factor kappa B kinase subunit epsilon | ENSG00000263528 |
|  | GJC1 | GJC1 gap junction protein gamma 1 | ENSG00000182963 |
|  | TFRC | Transferrin receptor | ENSG00000072274 |
|  | SLCO1B1 | Solute carrier organic anion transporter family member 1B1 | ENSG00000134538 |
|  | MBP | Myelin basic protein | ENSG00000197971 |
|  | S100B | S100 calcium binding protein B | ENSG00000160307 |
|  | MICA | MHC class I polypeptide-related sequence A | ENSG00000204520 |
|  | CPE | Carboxypeptidase E | ENSG00000109472 |
|  | SMARCA2 | SWI/SNF related, matrix associated, actin dependent regulator of chromatin, subfamily a, member 2 | ENSG00000080503 |
|  | EZH1 | Enhancer of zeste 1 polycomb repressive complex 2 subunit | ENSG00000108799 |
|  | SLC7A5 | Solute carrier family 7 member 5 | ENSG00000103257 |
|  | Cldn5 | claudin 5 | ENSG00000184113 |
|  | MFSD2A | MFSD2 lysolipid transporter A, lysophospholipid | ENSG00000168389 |
|  | NTN1 | Netrin 1 | ENSG00000065320 |
|  | CLDN1 | Claudin 1 | ENSG00000163347 |
|  | OCLN | Occludin | ENSG00000197822 |
|  | TJP1 | Tight junction protein 1 | ENSG00000104067 |
|  | PECAM1 | Platelet and endothelial cell adhesion molecule 1 (CD31) | ENSG00000261371 |
|  | DHH | Desert hedgehog signaling molecule | ENSG00000139549 |
|  | SHH | Sonic hedgehog signaling molecule | ENSG00000164690 |
|  | FOXO1 | Forkhead box O1 | ENSG00000150907 |
|  | HBB | Hemoglobin subunit beta | ENSG00000244734 |
|  | SPARC | Secreted protein acidic and cysteine rich | ENSG00000113140 |
|  | SLC16A1 | Solute carrier family 16 member 1 | ENSG00000155380 |
|  | CD36 | CD36 molecule | ENSG00000135218 |
|  | CLDN12 | Claudin 12 | ENSG00000157224 |
|  | IGF1R | Insulin like growth factor 1 receptor | ENSG00000140443 |
|  | LRP8 | LDL receptor related protein 8 | ENSG00000157193 |
|  | ABCC4 | ATP binding cassette subfamily C member 4 | ENSG00000125257 |
|  | SLC6A6 | Solute carrier family 6 member 6 | ENSG00000131389 |
|  | ABCG2 | ATP binding cassette subfamily G member 2 | ENSG00000118777 |
|  | JAM2 | Junctional adhesion molecule 2 | ENSG00000154721 |
|  | SLC39A8 | Solute carrier family 39 member 8 | ENSG00000138821 |
|  | SLC40A1 | Solute carrier family 40 member 1 | ENSG00000138449 |
|  | SLC22A8 | Solute carrier family 22 member 8 | ENSG00000149452 |
|  | ABCB1 | ATP binding cassette subfamily B member 1 | ENSG00000085563 |
|  | FCGRT | Fc gamma receptor and transporter | ENSG00000104870 |
|  | CTNNB1 | Catenin beta 1 | ENSG00000168036 |
|  | ABCC1 | ATP binding cassette subfamily C member 1 | ENSG00000103222 |
|  | GADD45GIP1 | GADD45G interacting protein 1 | ENSG00000179271 |
|  | SLCO2A1 | Solute carrier organic anion transporter family member 2A1 | ENSG00000174640 |
|  | INSR | Insulin receptor | ENSG00000171105 |
|  | MLC1 | Modulator of VRAC current 1 | ENSG00000100427 |
|  | PGM5 | Phosphoglucomutase 5 | ENSG00000154330 |
|  | SLC29A1 | Solute carrier family 29 member 1 | ENSG00000112759 |
|  | SLC2A1 | Solute carrier family 2 member 1 | ENSG00000117394 |
|  | SLC2A3 | Solute carrier family 2 member 3 | ENSG00000059804 |
|  | SLC2A14 | Solute carrier family 2 member 14 | ENSG00000173262 |
|  | SLC1A3 | Solute carrier family 1 member 3 | ENSG00000079215 |

| Supplementary Table 3. Predicted miRNAs for gene associated to glymphatic system and IL-1β. | | | | |
| --- | --- | --- | --- | --- |
| Index | **Targeted miRNAs** | **p-value** | **-log (p-value)** | **Genes** |
|  | mmu-miR-504-3p | 7.78E-08 | 7.109243748 | SLC4A10,CCR7,SLC9A1,AQP4,GFAP,PROX1 |
|  | mmu-miR-122b-5p | 3.95E-07 | 6.402963335 | VEGFC,DAG1,DMD,DTNA,PROX1 |
|  | hsa-miR-587 | 9.25E-07 | 6.033999142 | VEGFC,DMD,SLC12A2,SLC1A3,DTNA,GFAP |
|  | hsa-miR-122b-5p | 1.57E-06 | 5.805485658 | VEGFC,DAG1,DMD,AQP4,DTNA |
|  | hsa-miR-2053 | 1.69E-06 | 5.771856392 | SLC4A10,DMD,SLC12A2,AQP4,DTNA |
|  | hsa-miR-510-3p | 2.12E-06 | 5.673664139 | DAG1,DMD,AQP4,SLC1A3,DTNA,PROX1 |
|  | hsa-miR-499b-5p | 7.08E-06 | 5.150273556 | DMD,SLC12A2,AGRN,DTNA |
|  | mmu-miR-7240-5p | 8.12E-06 | 5.090337015 | CCR7,SLC9A1,CCL21,DTNA |
|  | hsa-miR-4328 | 1.11E-05 | 4.956637722 | SLC4A10,SLC12A2,AQP4,DTNA,GFAP,PROX1 |
|  | mmu-miR-7216-5p | 1.13E-05 | 4.946921557 | SLC9A1,CCL21,DTNA,GFAP |
|  | hsa-miR-4429 | 1.19E-05 | 4.925183559 | DAG1,CCR7,DMD,SLC12A2,DTNA |
|  | hsa-miR-320c | 1.20E-05 | 4.919373513 | DAG1,CCR7,DMD,SLC12A2,DTNA |
|  | hsa-miR-320d | 1.20E-05 | 4.919373513 | DAG1,CCR7,DMD,SLC12A2,DTNA |
|  | hsa-miR-320b | 1.20E-05 | 4.919373513 | DAG1,CCR7,DMD,SLC12A2,DTNA |
|  | hsa-miR-320a-3p | 1.20E-05 | 4.919373513 | DAG1,CCR7,DMD,SLC12A2,DTNA |

| Supplementary Table 4. Predicted miRNAs for gene associated to the blood-brain barrier and IL-1β. | | | | |
| --- | --- | --- | --- | --- |
| Index | **Targeted miRNAs** | **p-value** | **-log (p-value)** | **Genes** |
|  | hsa-miR-1277-5p | 4.81E-11 | 10.31830661 | SLC6A6,ABCC1,BTG2,SLC16A1,RGS5,CD36,SHH,SLC7A5,NTN1,CPE,SLCO1B1,UTRN,SLC2A1,SLC39A8,LRP8 |
|  | mmu-miR-6903-3p | 5.02E-10 | 9.299555899 | SLC6A6,S100B,IGF1R,IGF2,DHH,  SLC22A8,RGS5,SLCO2A1,PECAM1,INSR,SMARCA2,PGM5 |
|  | mmu-miR-466a-5p | 6.87E-10 | 9.162916849 | SLC6A6,SPARC,IGF2,SLC22A8,RGS5,  SLCO2A1,SMARCA2,SHH,PGM5 |
|  | mmu-miR-466p-5p | 6.87E-10 | 9.162916849 | SLC6A6,SPARC,IGF2,SLC22A8,RGS5,  SLCO2A1,SMARCA2,SHH,PGM5 |
|  | mmu-miR-1187 | 9.96E-10 | 9.00160987 | SLC6A6,SPARC,IGF2,SLC22A8,RGS5,  SLCO2A1,SMARCA2,SHH,PGM5 |
|  | mmu-miR-466l-5p | 3.91E-09 | 8.407490152 | SLC6A6,SPARC,IGF1R,IGF2,DHH,  SLC22A8,RGS5,SLCO2A1,PECAM1,INSR,SMARCA2,PGM5 |
|  | mmu-miR-466e-5p | 5.23E-09 | 8.281581358 | SLC6A6,SPARC,IGF2,SLC22A8,RGS5,SLCO2A1,SHH,PGM5 |
|  | mmu-miR-466k | 1.65E-08 | 7.783042793 | SLC6A6,SPARC,IGF1R,IGF2,DHH,  SLC22A8,RGS5,SLCO2A1,PECAM1,INSR,SMARCA2,PGM5 |
|  | mmu-miR-466i-5p | 1.65E-08 | 7.783042793 | SLC6A6,SPARC,IGF1R,IGF2,DHH,  SLC22A8,RGS5,SLCO2A1,PECAM1,INSR,SMARCA2,PGM5 |
|  | mmu-miR-466d-5p | 1.66E-08 | 7.780153614 | SLC6A6,SPARC,IGF1R,IGF2,DHH,SLC22A8,  RGS5,SLCO2A1,PECAM1,INSR,SMARCA2,PGM5 |
|  | hsa-miR-4650-3p | 3.07E-08 | 7.51243744 | CD36,INSR,OCLN,UTRN,CLDN12,CLDN1,SLC39A8 |
|  | mmu-let-7a-1-3p | 3.56E-08 | 7.449038248 | FOXO1,BTG2,IGF1R,TJP1,SMARCA2,CTNNB1,  UTRN,CLDN12,PGM5,LRP8 |
|  | mmu-let-7c-2-3p | 3.56E-08 | 7.449038248 | FOXO1,BTG2,IGF1R,TJP1,SMARCA2,CTNNB1,  UTRN,CLDN12,PGM5,LRP8 |
|  | mmu-miR-98-3p | 3.59E-08 | 7.445147566 | FOXO1,BTG2,IGF1R,TJP1,SMARCA2,CTNNB1,  UTRN,CLDN12,PGM5,LRP8 |
|  | mmu-let-7f-1-3p | 3.72E-08 | 7.42945706 | FOXO1,BTG2,IGF1R,TJP1,SMARCA2,CTNNB1,  UTRN,CLDN12,PGM5,LRP8 |

# **Supplementary Methods**

# **Supplementary Fig 1.** Schematic illustration of the protocol used for in vitro, in vivo, and bioinformatic experiments in the study. Stars (*) are indicted all treatments associated with TAA+HA130 PMI group. Abbreviation; CMI: Concurrent model intervention; inj: Injection; i.p.: Intraperitoneal; NS: Normal saline; PMI: Post model intervention; s.c.: Subcutaneous; TAA: Thioacetamide**.**

# **Immunofluorescence of GFAP in astrocyte cultures**

To characterize astrocytes, the expression of GFAP as a well-known indicator for astrocytes was visualized by immunocytochemistry method [1]. At first, seeded astrocytes in a 96-well plate were washed with PBS solution and fixed with 4% paraformaldehyde for 30 minutes at room temperature. Cell permeabilization was performed by incubating cells with 0.3% Triton X-100 (Sigma-Aldrich, Germany) for 5 minutes at 25°C. Non-specific binding sites were covered by 1% bovine serum albumin (BSA; Sigma-Aldrich, Germany) and 10% normal goat serum (Sigma-Aldrich, Germany) for 1 hour at 25°C. Next, cells were incubated overnight with a rabbit polyclonal antibody against GFAP (1:5000; ab7260, Abcam, USA). On the second day, cells were incubated with goat anti-rabbit fluorescent antibody (1:1000; ab6717, Abcam, USA) for 80 minutes at room temperature. To counterstain cell nuclei, propidium iodide (PI) was used for 20 seconds. The number of GFAP^+^ cells was observed with an inverted fluorescent microscope (Zeiss Axiovert 200, UK).

# **Cell viability assay**

To evaluate the effect of ammonia and HA130 on cell viability and cell proliferation, MTT assay (3-[4,5-dimethylthiazol-2-yl]-2,5 diphenyl tetrazolium bromide; Sigma-Aldrich, Germany) was performed as previously explained [2]. To do this, ~10,000 cells were seeded in a 96-well plate and 10µl of MTT solution was added to each well and then incubated at 37°C with 5% CO2 and 95% air for 4 hours. After incubation, supernatants were discarded and 100µl of dimethyl sulfoxide (DMSO; CARLO ERBA Reagents GmbH, Germany) was added and then shaken at 60 rpm for 1 hour at a 37°C shaker incubator. The absorbance was read at 570 nm and 630 nm in the microplate spectrophotometer (Epoch, Agilent Technologies, Inc; USA).

# **Flow cytometry and determination of cell volume changes**

Alternations in astrocyte cell volume were determined with a flow cytometer (BD FACSCalibur Flow Cytometer, Canada) and data were analyzed with FlowJo software (version 10.5.3) as previously described [3]. Briefly, astrocytes were immediately detached and suspended in a cold PBS solution for consequent analysis by flow cytometer. To estimate astrocyte volume, forward scatter (FSC) was measured that represents cell size.

# **Novel object recognition test and clinical stage of encephalopathy**

Novel object recognition test was used to evaluate recognition memory at the end of the experiment in mice. In the habituation phase, mice were placed into an empty open field box (45× 45 × 45 cm) to habituate with test arena for 5 minutes. Second day in the identical phase of training, mice were exposed for 10 minutes to two same objects that were placed similarly at a distance of 5 cm from the corners of the arena. The exploration time that each mouse spent around each object (touch object) was recorded manually by two different stopwatches. Six hours after familiarization, one of the identical objects was replaced with a novel object and mice were placed into the arena and exposed to objects for 10 minutes. Time spent around the familiar object and novel abject were separately recorded. After each trial, objects and the arena were cleaned with 70% ethanol. Time spent around the novel objects compared to familiar object was analyzed according to the following formula [4]:

Discrimination index = $\frac{(Time spent around novel object)-(Time spent around identical object)}{Total time spent around the objects}$

The stage of encephalopathy in an open field box was recorded twice a day as follows [5]: When the spontaneous activity was reduced and animals were agitated, mice were given grade I; Mice with grade II had mild ataxia; where spontaneous activity was lost but righting reflex was intact, mice were given grade III; In grade IV, mice were lost the righting reflex but pain reflex was intact; Coma was represented stage V.

# **Hematological analysis**

At the end of experimental period, mice were euthanized and blood samples were taken transcardially and stored in calcium ethylenediaminetetraacetic acid (EDTA)-containing tubes. Tubes were immediately sent to the laboratory for measurement of hemoglobin, hematocrit, red blood cells count, leukocytes count, platelets count, mean corpuscular volume (MCV), mean corpuscular hemoglobin (MCH), and mean corpuscular hemoglobin concentration (MCHC) using fully automated hematology analyzer (SYSMEX KX21, Japan).

# **Enzyme-linked immunosorbent assays**

The levels of LPA, and pro-inflammatory cytokines, such as interleukin-1 beta (IL-1β), interleukin-6 (IL-6), and Chemokine (C-C motif) ligand 3 (CCL3) were measured in animal serum, liver, cerebral cortex, and cell culture supernatant from astrocytes and levels of iPLA2 and tumor necrosis factor alpha (TNF alpha) were assayed in mice plasma, cerebral cortex, and liver tissues using some specific sandwich enzyme-linked immunosorbent assay (ELISA) kits according to manufacturer’s instruction (ZellBio, Germany; R&D system, USA; Karmania Pars Gene, Iran). Briefly, pre-weighted tissues were homogenized in ice-cold PBS solution and then centrifuged at 5500 rpm for 10 minutes at 4°C. Supernatants were collected and stored at -80°C for consequent analysis. All samples were read at 450 nm on an ELISA plate reader (Epoch, Agilent Technologies, Inc; USA) followed by LPA, iPLA2 and cytokines quantification.

# **Measurement of ammonia level**

To measure ammonia level, blood samples, liver tissue, and cortical pieces of frontal cortex were collected. Blood samples were collected in EDTA coated tube and centrifuged at 12000 rpm for 3 minutes at 4^°^C to separate plasma and subsequently stored on ice. Almost 100 mg of liver tissue and 50 mg of cerebral pieces were homogenized in 1000 µL and 300 µL of ice-cold phosphate buffer saline, respectively and centrifuged at 5500 rpm for 10 minutes at 4^°^C. All supernatants were harvested, liquated, and stored at -80^°^C for subsequent analysis. Ammonia levels in plasma and supernatants were measured by ammonia assay kits according to the manufacturer’s protocol (BXC0376, Biorexfars, Fars, Province, Iran) using an Epoch microplate spectrophotometer (Agilent Technologies, Inc; USA). According to the kit, ammonia reacts with α-Ketoglutarate and nicotinamide adenine dinucleotide phosphate (NADPH) in presence of L-glutamate dehydrogenase (GLDH). Ammonia concentration at 340 nm is proportional to a decrease in NADPH due to its oxidation.

# **Liver function tests and relative tissue weight**

To assay liver function, some important liver-specific proteins were evaluated. Immediately after decapitation of mice, blood samples were transcardially collected into the dry centrifuge tubes to obtain serum. Serum samples were stored at -20°C for subsequent biochemical analysis. The liver injury and failure were determined by measurement of some liver-specific proteins aspartate aminotransaminase (AST; BXC0205; Biorexfars, Iran), alanine aminotransaminase (ALT; BXC0215; Biorexfars, Iran), and alkaline phosphatase (ALP; BXC0187; Biorexfars, Iran) into the serum according to manufacturer's protocol using an automatic chemistry analyzer (BT1500; Biotecnica Instruments; Italy). Serum albumin (BXC0222; Biorexfars, Iran) was also measured according to the manufacturer's protocol. To calculate relative liver weight, the whole liver was carefully dissected and weighted. Below formula was used to compute relative liver weight [6]:

Relative liver weight (mg.g^-1^) = $\frac{Absolute liver weight}{Animal body weight}$

# **Cerebral edema**

The brain water content in all experimental groups was determined by the wet/dry weight method at the end of experiments [7]. Immediately after anesthesia (ketamine and xylazine) and decapitation, 10 mg of cortical samples were dissected from each animal and placed into a pre-weighed tube and were considered as wet weight. Tissues were dried at 120°C for 24 hours in an oven. Then, samples were weighted and dry weights were recorded. Finally, cerebral water content was calculated as a percentage using the following formula:

Percentage of water content = ($\frac{\left( Wet weight-Dry weight \right)}{Wet weight}$)×100

# **Tissue preparation for histopathology and immunohistochemistry**

Duodenum, colon, liver, and brain samples from different experimental groups were fixed in 10% neutral-buffered formalin (Dr Mojallali Industrial Chemical Complex Co. Iran) in normal saline for 72 hours and then were stored in 70% ethanol (Merck, USA). Slides were prepared by paraffin-embedded method, deparaffinized and rehydrated in xylene and ethanol, respectively, and further staining was performed as explained follows.

# **Hematoxylin and eosin staining for histopathology**

To evaluate cellular parameters, hematoxylin and eosin (H&E) staining was used for 5 µm tissue thickness of intestine, liver, and brain samples. Slides were prepared by paraffin-embedded method. Liver injury was evaluated in 5 fields of microscopic view in each section (lens ×40) and then the mean scores were considered as liver injury scores in each group as previously identified [8]. All scores were examined as follows: 0, Intact liver without pathological findings; 1, hepatocyte vacuolation and focal nuclear pyknosis; 2, moderate injury, severe nuclear pyknosis and cytoplasmic hypereosinophilia; 3, necrosis, hemorrhage and neutrophil infiltration [8]. The number of plasma cells, lymphocytes, and neutrophils was determined in lamina propria of duodenum and colon in 5 fields of microscopic view (lens ×100) in each section. Finally, the average number of cells was reported in each group. The number of degenerative cells in coronal sections of forebrain was also counted in 6 fields of microscopic view (lens ×40) in each section as previously described [9].

# **Periodic acid‑Schiff staining for intestines, liver and cerebral cortex samples**

To assess mucin-secreting cells in intestines, intracellular glycogen stores in liver and, number of active microglia/macrophage in cerebral cortex periodic acid‑Schiff (PAS) staining was performed by DDK kit (Italy) according to the manufacturer’s protocol. Briefly, slides were stained with periodic acid solution for 10 minutes, rinsed with distilled water for 1 minute, stained with Schiff reagent for 15 minutes, and treated 3 times with sodium metabisulphite solution for 2 minutes. Following rinsed with distilled water, sections were counterstained with Mayer haemallum solution for 10 minutes, rinsed with tap water for 5 minutes, de-hydrated through increasing ethanol gradient, cleared with xylene, and mounted for microscopic examinations. The number of duodenal PAS-positive cells, colon goblet cells (gel-forming mucins-secreting cells), and cerebral microglia/macrophages were counted in 7-8 fields of microscopic view (lens ×40) in each section. The average number of PAS-positive cells was reported in duodenum, colon[10], and frontal cortex [11] of each group.

# **Immunohistochemistry**

To visualize the distribution and morphology of astrocytes in the frontal cortex of mice, immunohistochemistry against GFAP was conducted. Coronal sections of frontal cortex were incubated in 100 °C PBS with pH ~ 7.4 for 25 minutes to conduct antigen retrieval. Cell permeabilization and blocking of the non-specific binding site were performed with 1% BSA (Sigma-Aldrich, Germany) and 2% triton X100 (Sigma-Aldrich, Germany), respectively. Endogenous peroxidase activity was suppressed by incubating sections with 3% hydrogen peroxide (CARLO ERBA Reagents GmbH, Germany) in methanol (TITRACHEM, Iran) for 15 minutes at 25 °C in darkness. The samples were also incubated for 45 minutes with normal goat serum (Sigma-Aldrich, Germany) as a blocking solution at 25 °C, then were incubated at 4°C overnight with a rabbit polyclonal antibody against GFAP (1:1000; ab7260, Abcam, USA) that was diluted with 1% BSA in PBS. The sections were washed in PBS and then incubated for 2 hours with goat anti-rabbit IgG-HRP (1:500; ab6721, Abcam, USA). The sections were stained with 3,3'-diaminobenzidine (1:40; ab64238, Abcam, USA) for 6 minutes, counterstained with hematoxylin for 3 minutes, de-hydrated through increasing ethanol gradient, and cleared with xylene and cover slipped for following microscopic examinations. The number of GFAP-positive cells was counted in 7 fields of microscopic view (lens ×40) and then the average number of GFAP-positive cells was reported in each group.

# **Blood-brain barrier permeability**

At the end of experiment, mice received 4 mL/kg of 2% Evans blue (Sigma-Aldrich, Germany) in normal saline under anesthesia with ketamine and xylazine [12]. Evans blue was injected through retro-orbital root. 30 minutes after injection of dye, mice were sacrificed, and cerebral cortex was collected. Almost 50 mg of cerebral cortex were weighted and manually homogenized in 250 µl of ice-cold 50% trichloroacetic acid (TCA; Sigma-Aldrich, Germany). Homogenized samples were centrifuged at 4^°^C at 9000 rpm for 20 minutes. Supernatants were plated duplicate in a 96-well plate and colorimetric assay was performed using a spectrophotometer (BioTek 800 TS, USA) at the absorptions of 620-630 nm.

# **Ultra-structural examination of the blood-brain barrier**

Transmission electron microscopy (TEM) was performed to examine the components of the blood-brain barrier (BBB) in the cerebral cortex of mice as previously described processes [13, 14]. Mice were sacrificed and cortical samples were dissected and fixed in 2.5% glutaraldehyde (TAAB Laboratories Equipment Ltd, UK) for 2 hours at 4°C, then post-fixed with 1% osmium tetroxide (TAAB Laboratories Equipment Ltd, UK) in 0.1 M phosphate buffer for 60 minutes, de-hydrated through increasing ethanol gradient, rinsed with propylene oxide (TAAB Laboratories Equipment Ltd, UK) and finally embedded in epoxy resin (TAAB Laboratories Equipment Ltd, UK). Then 70 nm sections were obtained on an ultramicrotome. Ultrathin sections were placed on copper grids (TAAB Laboratories Equipment Ltd, UK), stained with uranyl acetate (TAAB Laboratories Equipment Ltd, UK) and lead citrate and samples with1 mm^2^ thick was prepared for 120kb high voltage transmission electron microscope (ZEISS, LEO912AB, Germany). All photos were acquired from perivascular spaces, which contained the BBB structures as endothelial cells, pericyte, astrocyte end-feet and surrounding cortical neurons.

# **Ribonucleic acid extraction, complementary deoxyribonucleic acid synthesis and quantitative reverse transcriptase real-time polymerase chain reaction**

The messenger ribonucleic acid (mRNA) expression profile of LPA receptors, ectonucleotide pyrophosphatase/phosphodiesterase 2 (ENPP2; ATX gene) and aquaporin-4 (AQP4) in astrocytes, frontal cortex and liver (except AQP4) was determined by reverse transcription quantitative real-time polymerase chain reaction (RT-qPCR). Total RNA was extracted from astrocyte cultures, liver, frontal cortex and salivary glands using super RNA extraction kit (YT9080, Yektatajhiz Azma, Iran) according to the manufacturer’s instructions. complementary deoxyribonucleic acid (cDNA) synthesis was conducted from 1ug total RNA (was measured by a nanodrop spectrophotometer; Thermo Fisher Scientific, USA) using Oligo (dT)18 and Random hexamer primers, RNasin, dNTP and M-MLV (YT4500, Yektatajhiz Azma, Iran) through a thermal cycler (FlexCycler, Analytik Jena GmbH, Germany). Subsequently, RT-qPCR proceeded using synthesized cDNA, gene-specific primers (Supplementary Table 5; Pishgam biotech co, Iran) and SYBR Green qPCR (S054, Yektatajhiz Azma, Iran) through a LightCycler 96 (Roche, Switzerland) under condition as follows: preincubation at 95°C for 900 seconds, two-step amplification by 40 cycles of 95°C for 30 seconds and 60°C for 60 seconds and melting. β-actin (ACTB) and glyceraldehyde 3-phosphate dehydrogenase (GAPDH) were used as internal references for RT-qPCR. All results of TAA mice and NH_4_Cl-exposed astrocytes were expressed as relative fold change compared to sham/control groups.

| Supplementary Table.5 Real-time PCR primer information. | | |
| --- | --- | --- |
| Index | **Gene** | **Sequence (5′-3′)** |
|  | MUS ACTB F | AGGGAAATCGTGCGTGACAT |
|  | MUS ACTB R | GAACCGCTCGTTGCCAATAG |
|  | MUS AQP4 F | GCCTTTGTTGTGTGATGTTGAC |
|  | MUS AQP4 R | GATTGTCTGTTTGGCTCTCTGG |
|  | MUS ENPP2 (ATX) F | TCCCTACCCACTACTACAGCATC |
|  | MUS ENPP2 (ATX) R | AGACACAGAGAGAGGACCATCAC |
|  | MUS GAPDH F | CAACGACCCCTTCATTGACC |
|  | MUS GAPDH R | CTTCCCATTCTCGGCCTTGA |
|  | MUS LPAR1 F | GATGTTTCCCAATAACCCAGAC |
|  | MUS LPAR1 R | GGATTCACTCTGATGCTTGATG |
|  | MUS LPAR2 F | GCCACCTTAGTCAACATAGCAG |
|  | MUS LPAR2 R | AGAACAAGATGCCCAGTGAGAC |
|  | MUS LPAR3 F | AGAGATGAGCAGTGGGTTCC |
|  | MUS LPAR3 R | AATGGTGGCAAGATGGTGAC |
|  | MUS LPAR4 F | GCGAGTTGCCAGTTTACACG |
|  | MUS LPAR4 R | AGTGCCCAAGAAAGAGTGTGC |
|  | MUS LPAR5 F | GACCTTGTTGTTCCCTACGATG |
|  | MUS LPAR5 R | GCTGTTGGTAGAAGTCGTGTTG |
|  | MUS LPAR6 F | ATCCCTTAAACGACTGCCTATC |
|  | MUS LPAR6 R | TCCTCGGGTACTTCTTCCTTC |

| Supplementary Table.6 List of used antibodies | | | | |
| --- | --- | --- | --- | --- |
| Name | **Citation** | **Supplier** | **Cat no.** | **Clone no.** |
| Rabbit polyclonal to GFAP |  | Abcam | ab7260 | - |
| Goat Anti-Rabbit IgG H&L (FITC) |  | Abcam | ab6717 | - |
| Goat Anti-Rabbit IgG H&L (HRP) |  | Abcam | ab6721 | - |

| Supplementary Table.7 List of used organisms | | | | | | |
| --- | --- | --- | --- | --- | --- | --- |
| Name | **Citation** | **Supplier** | **Strain** | **Sex** | **Age** | **Overall n number** |
| Mice |  | Iran University of Medical Sciences | C57B6/J | Neonate for astrocytes extraction | 1-3 days | 18 |
| Mice |  | Iran University of Medical Sciences | C57B6/J | Male | 8-10 weeks | 122 |

| Supplementary Table.8 List of used software | | |
| --- | --- | --- |
| Software name | **Manufacturer** | **Version** |
| CorelDRAW | Corel Corporation | Version 2020 |
| Prism | GraphPad | Version 8.3.0 |
| FlowJo | BD Bioscience | Version 10.5.3 |
| Excel | Microsoft | Version 2016 |
| Gen5 | BioTek | Version 2.09 |
| LightCycler® 96 | F. Hoffmann-La Roche Ltd | SW 1.1 |
| Adobe Photoshop | Adobe Systems | Version 7 |

| Supplementary Table.9 List of used agent/drug | | |
| --- | --- | --- |
| Reagent/ drug name | **Company** |  |
| Thioacetamide | Sigma-Aldrich |  |
| HA130 | Cayman Chemical |  |
| Ammonium chloride | TITRACHEM |  |
| 3-[4,5-dimethylthiazol-2-yl]-2,5 diphenyl tetrazolium bromide (MTT) | Sigma-Aldrich |  |

# **Supplementary References**

1. Shi Z, Zhang W, Lu Y, Lu Y, Xu L, Fang Q, et al. Aquaporin 4-Mediated Glutamate-Induced Astrocyte Swelling Is Partially Mediated through Metabotropic Glutamate Receptor 5 Activation. Frontiers in cellular neuroscience 2017, 11: 116.

2. Corvace F, Faustmann TJ, Faustmann PM, Ismail FS. Anti-inflammatory properties of lacosamide in an astrocyte-microglia co-culture model of inflammation. European journal of pharmacology 2022, 915: 174696.

3. Zholos A, Beck B, Sydorenko V, Lemonnier L, Bordat P, Prevarskaya N, et al. Ca(2+)- and volume-sensitive chloride currents are differentially regulated by agonists and store-operated Ca2+ entry. The Journal of general physiology 2005, 125(2): 197-211.

4. Lueptow LM. Novel Object Recognition Test for the Investigation of Learning and Memory in Mice. Journal of visualized experiments : JoVE 2017(126): 55718.

5. Jayakumar AR, Bethea JR, Tong XY, Gomez J, Norenberg MD. NF-κB in the mechanism of brain edema in acute liver failure: studies in transgenic mice. Neurobiology of disease 2011, 41(2): 498-507.

6. Stefanello ST, Flores da Rosa EJ, Dobrachinski F, Amaral GP, Rodrigues de Carvalho N, Almeida da Luz SC, et al. Effect of diselenide administration in thioacetamide-induced acute neurological and hepatic failure in mice. Toxicology Research 2015, 4(3): 707-717.

7. Rama Rao KV, Verkman AS, Curtis KM, Norenberg MD. Aquaporin-4 deletion in mice reduces encephalopathy and brain edema in experimental acute liver failure. Neurobiology of disease 2014, 63: 222-228.

8. Chen T-M, Subeq Y-M, Lee R-P, Chiou T-W, Hsu B-G. Single dose intravenous thioacetamide administration as a model of acute liver damage in rats. Int J Exp Pathol 2008, 89(4): 223-231.

9. El-Baz FK, Elgohary R, Salama A. Amelioration of Hepatic Encephalopathy Using <i>Dunaliella salina</i> Microalgae in Rats: Modulation of Hyperammonemia/TLR4. BioMed Research International 2021, 2021: 8843218.

10. Xie S, Zhang H, Matjeke RS, Zhao J, Yu Q. Bacillus coagulans protect against Salmonella enteritidis-induced intestinal mucosal damage in young chickens by inducing the differentiation of goblet cells. Poultry Science 2022, 101(3): 101639.

11. Idol RA, Wozniak DF, Fujiwara H, Yuede CM, Ory DS, Kornfeld S, et al. Neurologic Abnormalities in Mouse Models of the Lysosomal Storage Disorders Mucolipidosis II and Mucolipidosis III γ. PLOS ONE 2014, 9(10): e109768.

12. Grant S, McMillin M, Frampton G, Petrescu AD, Williams E, Jaeger V, et al. Direct Comparison of the Thioacetamide and Azoxymethane Models of Type A Hepatic Encephalopathy in Mice. Gene expression 2018, 18(3): 171-185.

13. Ampawong S, Chaisri U, Viriyavejakul P, Nontprasert A, Grau GE, Pongponratn E. Electron microscopic features of brain edema in rodent cerebral malaria in relation to glial fibrillary acidic protein expression. Int J Clin Exp Pathol 2014, 7(5): 2056-2067.

14. Faleiros BE, Miranda AS, Campos AC, Gomides LF, Kangussu LM, Guatimosim C, et al. Up-regulation of brain cytokines and chemokines mediates neurotoxicity in early acute liver failure by a mechanism independent of microglial activation. Brain research 2014, 1578: 49-59.
