# Supplementary figures and images for: Inhibition of autotaxin alleviates pathological features of hepatic encephalopathy at the level of gut–liver–brain axis: an experimental and bioinformatic study
Source: Cell Death Dis. 2023 Aug 1;14(8):490. doi: 10.1038/s41419-023-06022-5 (PMC10394058; doi:10.1038/s41419-023-06022-5)

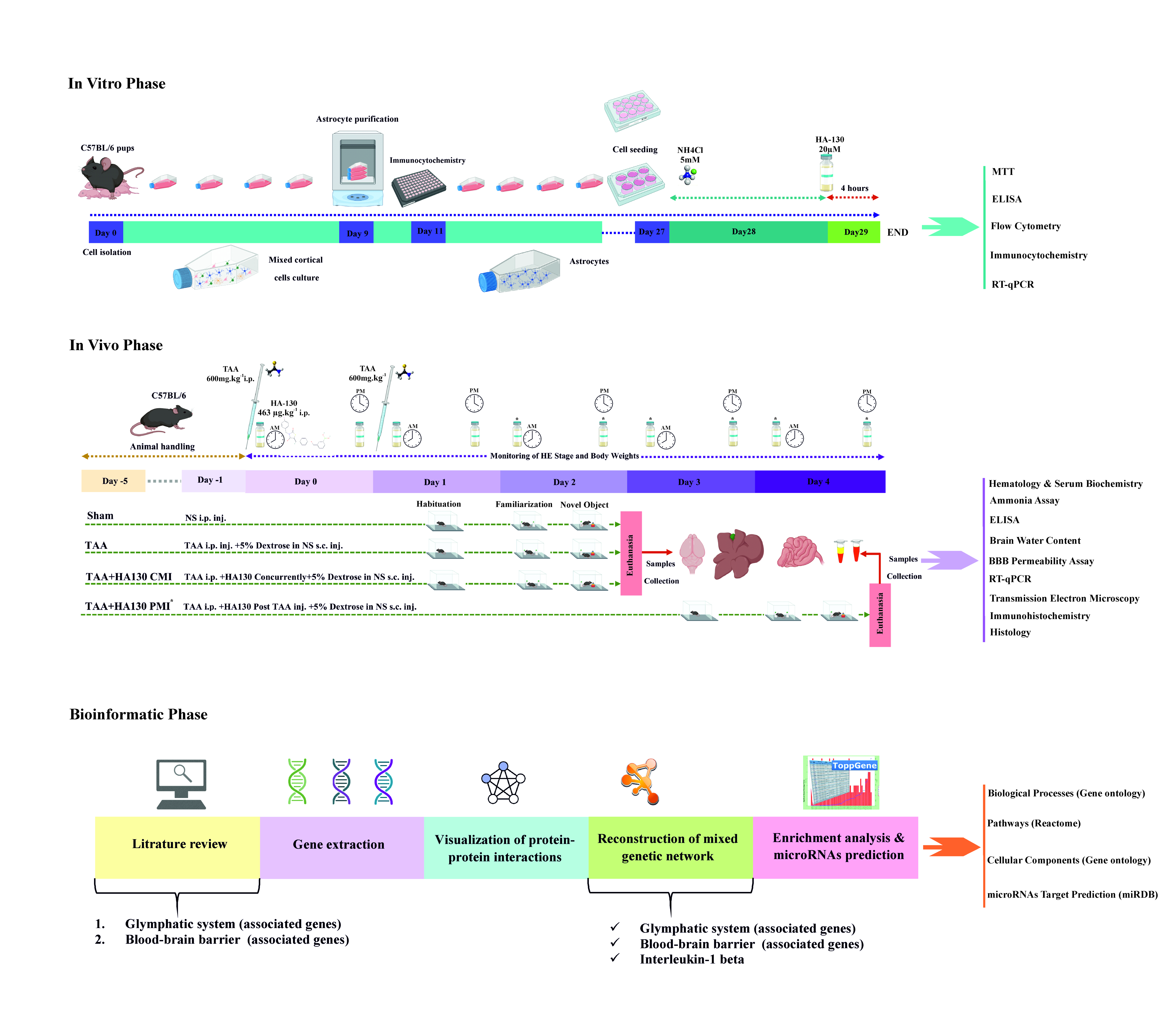

Supplement: Supplementary file 2 — Supplementary Figure 1 [file 41419_2023_6022_MOESM2_ESM.jpg]
